# Multiscale investigation of pore structure heterogeneity in carbonate rocks using digital imaging and SCAL measurements: A case study from Upper Jurassic limestones, Abu Dhabi, UAE

**DOI:** 10.1371/journal.pone.0295192

**Published:** 2024-02-08

**Authors:** Hind Sulieman, Mohamed Soufiane Jouini, Mohammad Alsuwaidi, Emad W. Al-Shalabi, Osama A. Al Jallad

**Affiliations:** 1 Department of Earth Sciences, Khalifa University of Science and Technology, Abu Dhabi, United Arab Emirates; 2 Department of Mathematics, Khalifa University of Science and Technology, Abu Dhabi, United Arab Emirates; 3 Department of Chemical and Petroleum Engineering, Research and Innovation Center on CO_2_ and Hydrogen, Khalifa University of Science and Technology, Abu Dhabi, United Arab Emirates; 4 Halliburton Service in the Middle East and Asia Pacific Areas, Abu Dhabi, United Arab Emirates; China University of Mining and Technology, CHINA

## Abstract

This study presents a comprehensive analysis of rock properties for a selected group of six carbonate reservoir rock samples revealing complex structures at various length scales. Experimental laboratory methods as well as image analysis techniques were conducted in this study to characterize the macro- and micro-pores in mud- and grain-dominated limestones samples from the Upper Jurassic Arab Formation (Arab D member). Mercury Injection Capillary Pressure (MICP), porosimeter, and permeameter lab measurements were employed to assess the pore network heterogeneity and complexity. In addition, a multiscale rock imaging approach was implemented to detect grain and pore phases at several length scales using Thin Sections (TS), Scanning Electron Microscopy (SEM), Focused Ion Beam Scanning Electron Microscopy (FIB-SEM), as well as 3D X-ray Computed Tomography (CT), and micro-computed tomography images (MCT). Furthermore, the multifractal analysis method was applied on the MICP and FIB-SEM to characterize quantitatively the heterogeneity of the pores in the studied samples. Heterogeneous samples 3R, 4M, 5W, and 6M display the highest non-uniformity degree Δα values, falling within the range of [1.21, 1.39] based on FIB-SEM images. Samples 1G, 2R, 3R, and 5W exhibit more heterogeneous pore structure, with Δα values ranging from 0.73 to 1.49 based on the MICP experiments. The results and findings confirm the effectiveness of multifractal parameters Δα and the asymmetry degree in the vertical axis Δf(α) in quantifying and characterizing rock heterogeneity.

## 1. Introduction

Carbonate rocks are highly heterogeneous with variable pore geometry at several length scales due to depositional and diagenetic processes, which influence porosity and permeability [1–5]. Researchers have proposed different definitions for microporosity based on the different techniques and tools used to fit their research purposes. However, the most recent and accepted definition in literature, which will be adopted in this study, characterizes a micro-pore as a pore with size less than 10 μm in diameter and pore throats less than 0.5 μm [6–10]. Microporosity can occur in matrix, within grains, and between cements. The distribution of microporosity is based on morphometry and crystallometry of the micrite particles [4,11,12].

Digital rock physics (DRP) has been broadly used to assess pore size distribution, porosity, permeability, elasticity and other rock properties in porous media such as carbonate and siliciclastic rocks [13–15]. The main steps of the DRP method include: (1) imaging the rock sample using X-ray computed tomography (CT) or micro-computed tomography (MCT) scanners to produce 3D images at different resolutions, where each voxel represents a grey level derived from variable rock density. (2) Segmenting images to separate the grains and pores using image analysis techniques. (3) Evaluating the petrophysical properties using numerical simulation algorithms based on the segmented images [5,14,16]. In carbonate rocks, a multi-scale analysis is needed to better understand the influence of pore geometry on petrophysical properties. Three-dimensional imaging methods such as X-ray computed and micro-computed tomography allow exploring rock samples at several length scales from the centimeter down to the micrometer [14,17,18].

Utilizing this estimation on a standard core plug with an approximate diameter of 2.5 cm poses challenges. Achieving the necessary level of resolution to capture micropores while also ensuring a sufficiently broad field of view to accurately represent the macropores within the core plug is essential. However, it is not possible to capture any pore with size smaller than 20 μm in the 3D MCT while scanning the whole core plug due to detector size limitation [14]. Multiscale imaging serves as a reliable solution for addressing this issue by enabling the characterization of the pore network at various resolutions.

This paper implements experimental techniques, image acquisition, processing, and analysis to characterize the macro- and microporosity of six core plug samples from mud-dominated and grain-dominated limestone reservoir, Abu Dhabi, United Arab Emirates (UAE). The primary novelty in this study lies in the integration of experimental and digital image datasets across multiple length scales, which involves the application of multifractals in both forms of analysis.

## 2. Geological setting of the Arab Formation

The offshore oilfields in Abu Dhabi are situated eastward of the Arabian platform, located between two major structural highs; the Oman mountains to the northeast and Qatar Arch to the west. The area has been subjected to extensive subsidence during post-Lower Permian history that resulted in thick sedimentary section accumulation. The structural development in the area is dominated by large and simple folds. These folds are noted as expressions of deep-seated basement faults [19,20].

Furthermore, salt tectonics have a significant role in structure modification and development. The structural development of most Abu Dhabi offshore fields resulted from basement tectonics or/and the Hormuz salt movements during the Late Cretaceous. Generally, the salt has reached the surface on the west side of Abu Dhabi, carrying up with it enormous igneous and sedimentary sediments, which end up in forming islands, such as Das, Dalma, and Zirku. However, in other places, the salt is buried deeply, building up structures such as Zakum, Umm Shaif and Arzanah. Arzanah Field has a N-S elongated anticline structure with a moderate relief feature. The field is located in western side offshore Abu Dhabi specifically; 4 Kilometres south of Arzanah Island, 22 kilometres north of Jabal Dhanna and 24 Kilometres Southwest of Das island [21,22]. Maximum oil generation peak was reached into the Jurassic reservoir rocks of the Arab Formation during the Eocene from of Hanifa Formation [23].

**Fig 1:**
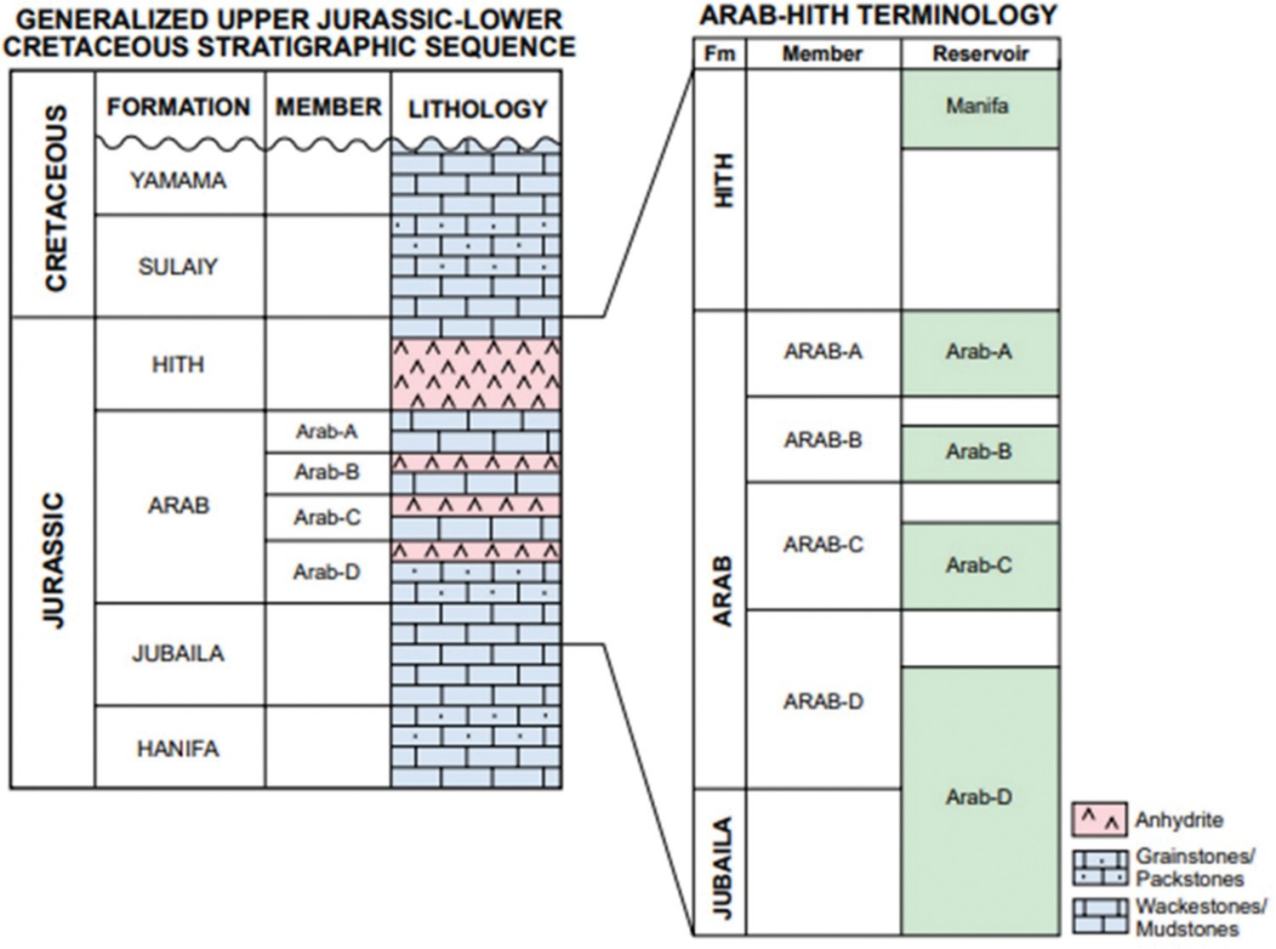
Simple stratigraphic column of Mesozoic represents some formations in the Eastern Arabian Peninsula [6].

The Arab Formation was deposited during Kimmeridgian to Tithonian periods and is divided into four main members labeled from bottom to top as Arab D, C, B, and A (**Fig 1**). Arab members are composed of shallow marine limestones and dolostones, and anhydrite with an average good porosity (~20%), and moderate-to-poor permeability (~0.01–10 mD) [24]. Arab B and A members are composed mainly of non-reservoir rocks. Arab D reservoir is the main target in this study, and it is divided into five sub-zones (D5, D4, D3, D2 and D1). On the other hand, Arab C is divided into three sub-zones (C3, C2, and C1). The Arab Formation is overlying Diyab Formation and is covered by the regional seal of the Hith Formation which is composed of evaporites and subordinate dolostones [25,26].

## 3. Materials and methods

**Fig 2:**
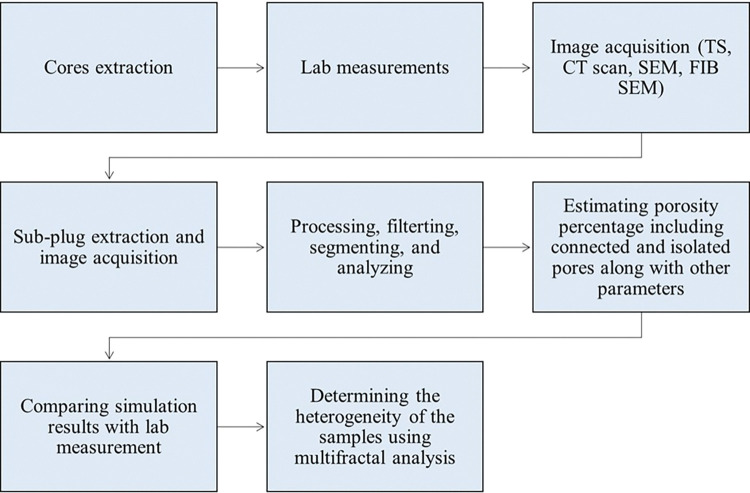
Micro- and macro-pores characterization workflow based on experimental and digital methods.

This section presents a comprehensive workflow on the pore geometry characterization implemented in this study (**Fig 2**). The experimental methods subsection includes the cores examination and lab measurements of gas porosity and permeability along with pore throat distributions. The image acquisition subsection includes different methods used to acquire multi-scale images such as computed tomography (CT), thin section (TS), and scanning electron microscopy (SEM). Finally, data processing and analyzing subsection include image filtering, segmentation, and integration to analyze the rock heterogeneity. The dataset employed in this research was collaboratively obtained by both Abu Dhabi National Oil Company (ADNOC) and Khalifa University (KU).

### 3.1 Experimental methods

#### 3.1.1 Core descriptions

**Fig 3:**
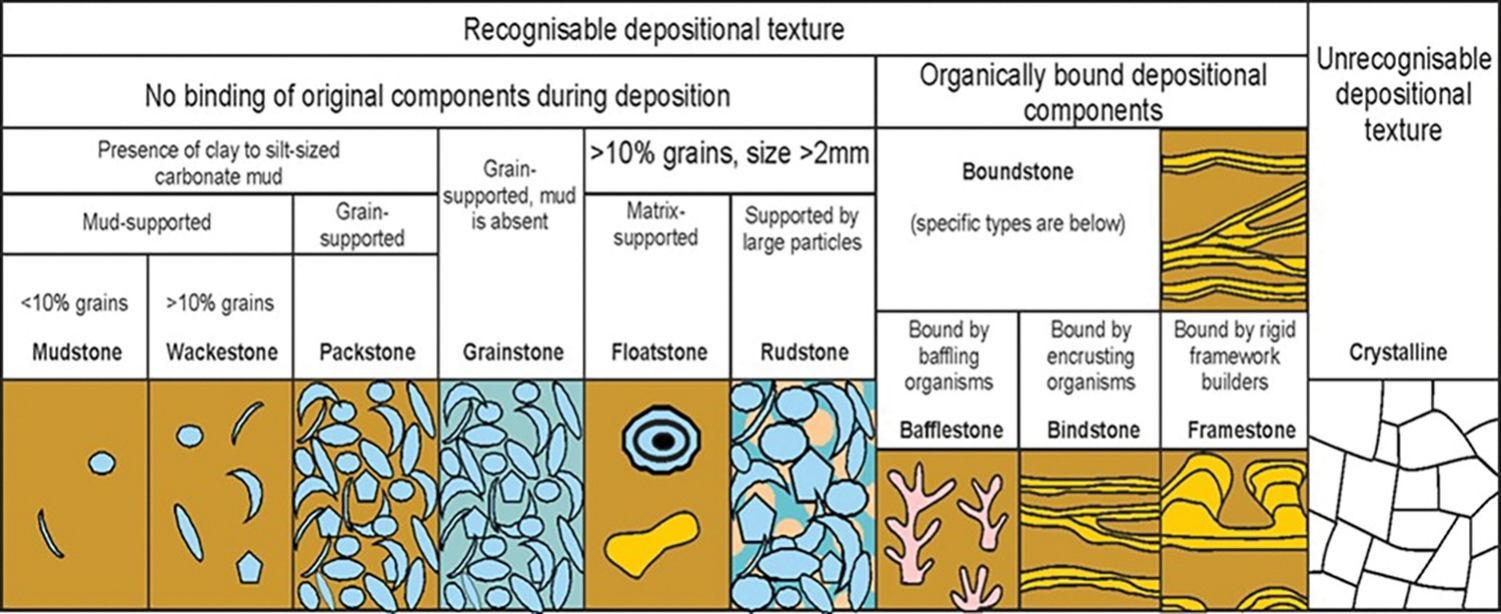
Classification for limestone textures [27,28].

Four hundreds sixty-six feet of slabbed cores from two wells, offshore Arab D were examined in terms of the lithology, textures, and grain types. Rocks were classified using the carbonate depositional texture classifications of Dunham (1962) and its later modification by Embry and Klovan (1972) (**Fig 3**). Six representative samples were selected for detailed analysis.

#### 3.1.2 Gas porosity and permeability measurements

Six representative one-inch plug samples were selected and extracted from the cores for high-resolution imaging. The plugs were selected from grain-dominated and mud-dominated limestones in Arab D in order to characterize the differences in pore networks between the muddy and grainy facies. All extracted samples were cleaned under refluxing hot solvents in Soxhlet extractors using toluene (C_7_H_8_) to remove all the hydrocarbons hosted within the pore systems. This was followed by methanol (CH_3_OH), which was used to remove all salts invaded into the pore system of the selected samples. After that, gas porosity and horizontal gas permeability were measured using helium porosimeter and nitrogen permeameter, respectively.

Porosity values provide estimation of the maximum amount of hydrocarbons that can be hosted in the reservoir. Porosity is calculated by dividing the pore volume by the bulk volume (**Table 1**). The bulk volume refers to the unit volume of the reservoir rock, comprising both the total pore volume and grain volume. To measure the grain volume of the extracted plugs, a calibrated helium gas volume expansion meter was used. Initially, the porosimeter was checked for potential leaks by testing the expansion with a steel blank in the matrix cup. The apparatus was then calibrated using five stainless steel discs of known volumes to establish a linear relationship between pressure and volume. The coefficient of determination of 0.9999 was obtained, indicating a strong linear relationship. After the calibration, the grain volumes were measured. The plug was placed into the matrix cup, and to fill any voids and minimize potential errors, a stainless-steel disc of known volume was added. Helium was then expanded into the matrix cup, and readings were taken once the transducer reading stabilized. Throughout the procedure, a constant temperature was maintained to ensure accuracy.

**Table 1:** Porosity, bulk volume, and horizontal permeability equations along with their descriptions.

Equation	Description
$Porosity=\frac{P_{v}}{B_{v}}*100\%$ (1)	P_v_ = pore volume in cc = (B_v_—G_v_) B_v_ = bulk volume in cc G_v_ = grain volume in cc calculated using a calibrated helium expansion porosimeter (Boyle’s Law)
$Bulk\;volume=\frac{Mass\;of\;mercury\;displaced}{Density\;of\;mercury}$ (2)	bulk volumes are calculated by immersing the core plug in mercury and the volume of mercury displaced by the sample is determined gravimetrically (Archimedes’ principle)
$Horizontal\;gas\;permeability\;(Kg)=\left(2000\frac{Q\mu P_{A}Lm}{A[{(P}_{1}+P_{A}{)}^{2}-(P_{2}+P_{A}{)}^{2}]}\right)$ (3)	Q = flow rate = DV/DT (cm^3^/s) L = length of sample in cm D = diameter of sample in cm A = cross sectional area of sample in cm^2^ = (D/2)^2^ × π μ = viscosity of gas in centipoise (at known temperature) P_A_ = atmospheric pressure in atm P_1_ = corrected upstream pressure in atm P_2_ = corrected downstream pressure in atm m = dynamic viscosity of the fluid (centipoise)

Gas permeability was measured using calibrated steady state permeameter with nitrogen gas as the flowing medium. The flow was allowed to stabilize before the readings were taken. The Hassler pressure for sealing the samples was 400 psig. To verify the permeameter’s operation, a full set of check plugs of known permeability were tested at the beginning of each run. After performing a confirmatory leak test, then the studied plugs were analyzed. The horizontal gas permeability was calculated using Darcy’s law (**Table 1**). The studied samples along with porosity and permeability values are summarized in **Table 2**.

**Table 2:** Measured and processed porosity and permeability of the studied plugs in Arab D limestones.

Sample	Depth (ft)	Plug Diameter (in)	Φ_He_ (%)	K_g_ (mD)	K_Hg_ (mD)	Φ_Hg_(%)
1G	10,838	1	12.9	4.60	3.8	10
2R	10,854	1	14.6	272	329.3	13
3R	10,861	1	17.6	230	33.2	9
4M	11,039	0.8	1.6	0.02	0	3
5W	10,916	0.8	6.8	0.05	0	4
6M	10,945	0.8	4.3	0.08	0	3

Φ_He_: *H*elium porosity, K_g_: *G*as permeability, K_Hg_: *P*ermeability obtained from MICP, Φ_Hg_: *P*orosity obtained from MICP.

#### 3.1.3 Mercury Injection Capillary Pressure (MICP)

Mercury injection capillary pressure (MICP) was conducted on six plug trims to determine the pore throat size distribution (PTSD) in grain-dominated and mud-dominated limestones. All extracted MICP trims were weighed and loaded into the selected penetrometers. These penetrometers were installed into different pressure ports, which allowed measuring the force needed to push a metal rod of known diameter into a growing medium [29]. Mercury injection experiment was used to force mercury under different known pressures throughout the evacuated pores in the studied trims. As the injected pressure increases gradually, the mercury enters progressively smaller pore throats starting from the well-connected macropores (>5 μm), mesopores (0.5–5 μm), and ultimately micropores (< 0.5 μm) [12,30]. An additional pore volume accessible through pore throats in a specified size range is represented by each volume increment injected. The various pore throat size distributions of the samples can be presented by plotting the volume of injected mercury versus the different pressure steps.

#### 3.1.4 Multifractal theory on MICP

Multifractal parameters are broadly used in earth sciences for analyzing the statistical heterogeneity of shale, sandstone, and carbonate rocks [31–33]. For example, Guan *et al*. (2020) assessed the degree of pore size heterogeneity throughout examining the width of singularity spectra from multifractal analysis derived from mercury intrusion in the unconventional shale reservoirs [34]. The results indicated that an increase in the width of singularity spectra corresponds to greater heterogeneity in pore size distributions. Song *et al*. (2018) implemented multi-fractal analysis on experimental MICP measurements to characterize the pore structures of the Upper Paleozoic tight sandstone reservoir [35]. They observed the presence of micro-fractures, which were the major factor influencing gas charging into the tight formation. Multifractal analysis examines self-similarities in objects geometries by detecting the presence of power-law scaling across different scales for various statistical moments. The primary benefit of employing multifractals is to offer quantitative measures of data heterogeneity and complexity. The main quantitative parameters estimated are the generalized dimensions (*D*_*q*_) and the mass exponent (*τ*_*q*_) defined by Equations (4) and (5), respectively (**Table 3**). In objects with homogeneous structures, the mass exponent (*τ*_*q*_) shows a linear correlation with moments *q*. On the other hand, in heterogeneous rocks, the slope of *τ*_*q*_ may deviate with respect to *q* and this variation is related to heterogeneity degree. The box counting (BC) technique is the most common method used to determine multifractal parameters. This method requires uniformly spaced data measurements with a scale size ε. The acquired MICP measurements were irregularly spaced in this study, so linear interpolation was applied to obtain regularly spaced points data divided into sub-intervals of *N* = 2^*10*^ = 1024.

**Table 3:** Numerical equations used to study the rock structure heterogeneity using MICP and FIB-SEM dataset.

Equation	Description
$D_{q}=\frac{1}{q-1}\underset{\varepsilon \to 0}{\lim}\frac{log\sum_{k}P_{k}^{q}(\varepsilon )}{log\varepsilon }$ (4)	*P*_*i*_(*r*)∝*r*^α^ *D*_*q =*_ generalized dimension
$\tau_{q}=(q-1)D_{q}$ (5)	*τ*_*q*_ = mass exponent
$f(\alpha )=q\alpha -\tau_{q}$ (6)	*f*(*α*) = singularity spectrum
$\alpha =\frac{d\tau_{q}}{dq}$ (7)	*α* = *singular exponen*
$D_{0}=\underset{\varepsilon \to 0}{\lim}\frac{ln(N(\epsilon ))}{ln(\frac{1}{\epsilon })}$ (8)	D_0_ = fractal dimension
$P_{k}(\varepsilon )∼\varepsilon^{\alpha_{k}}$ (9)	*ε =* the box size, *α*_*k*_ = the Lipschitz–Holder exponent characterizing the singularity strength in the *k*^th^ box

Furthermore, the multifractal theory is based on a relationship between singular exponent *α* and singularity spectrum *f*(*α*) as shown in **Table 3**, Equations (6) and (7), which is discussed later in the paper. The *Δα* = *α*_*min*_-*α*_*max*_ parameter represents the degree of pore structures non-uniformity. The high *Δα* values reflect large spatial complexity data. The singularity spectrum curve *f*(*α*) symmetry provides a quantitative heterogeneity assessment of the data, where heterogeneous data reveals asymmetry curves, while uniform-homogeneous data shows symmetric curves [36]. The parameter refers to the characteristics of singularity spectra shape. The shape characteristics of singularity spectra *f*(*α)* are represented by the parameter *Δf* = *f*(*α*_*min*_)−*f*(*α*_*max*_). The curve *f*(*α)* exhibits either a right or left asymmetry, indicating the dominance of a particular probability subset [37].

### 3.2 Image acquisition

#### 3.2.1 CT scanning

Six representative samples (**Table 2**) were selected from mud-dominated and grain-dominated limestones for CT-scan imaging using Toshiba Aquilion scanner. A dual energy CT scanning (DECT) method involves scanning samples with X-ray beams at two distinct energy levels (Siddiqui and Khamees, 2004). The resulting three-dimensional images of the scanned core samples at large scale have a resolution of 0.5 mm/voxel. The sample’s atomic number, photo electric factor (PEF), and bulk density (ρ_b_) can be derived from the energy level information [38,39]. By analyzing CT numbers and deriving the bulk density, it becomes possible to qualitatively assess the uniformity of porosity within the sample. The effective atomic number offers quantitative insights into the mineralogical variations within the samples. Additionally, representative CT images aid in identifying key heterogeneities in micro-structures, textures, fabrics, and density fluctuations within core samples. These observations serve as a guide for selecting specific locations of sub-samples for subsequent higher resolution scans, as well as for conducting MICP, scanning electron microscopy (SEM), and focused ion beam scanning electron microscopy (FIB-SEM) analyses.

The MCT acquisition was performed using the Zeiss Versa XRM-500 scanner. First, all samples were scanned with a resolution of 27 μm/voxel by rotating each scanned sample 360 degrees inside the MCT scanning machine. The visual inspection of sample allowed identifying main heterogeneities related to variability in pore network. Therefore, MICP was used as a guide for the optimal extraction size of subsets and image scanning resolution. A physical extraction of subsets was carried out to investigate the macro- and micro-porosities through a series of multi-resolution imaging [14,17,18,39–41].

#### 3.2.2 Thin section (TS)

**Fig 4:**
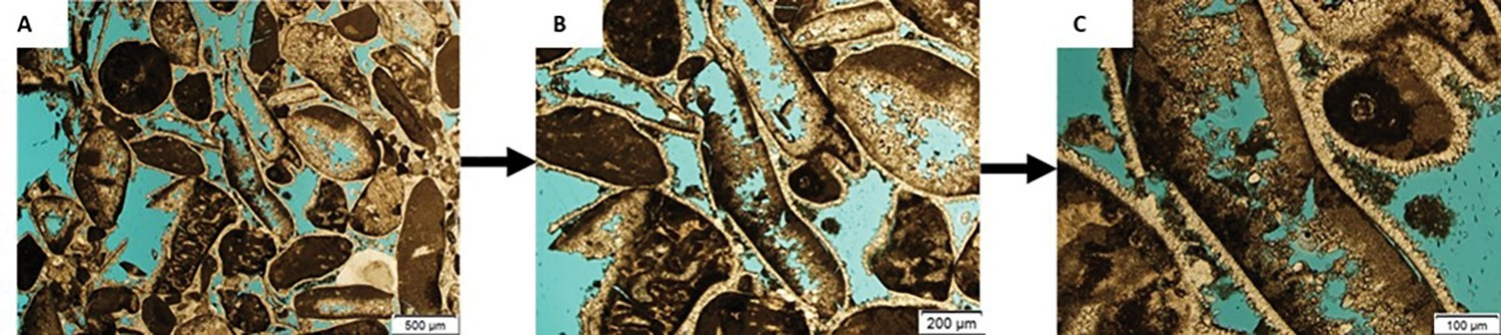
Optical photomicrographs at plain polarized light (PPL) showing the intergranular (between micritized grain) and intragranular (within micritized grain) porosities at different magnifications of (A) 2x, (B) 4x, and (C) 10x.

Eighty-two representative thin sections from Arab D limestones were examined by optical microscope. The 2D petrography allows identification of the porosity evolution, and succession of diagenetic phases occurred in the area, e.g. calcite cementation and dissolution, which affect the quality of porosity and permeability. The samples were cut and prepared with a thickness of 30 μm (0.03 mm). Thin sections were impregnated with blue epoxy and Alizarin-Red-S solution at high pressure. The injected blue epoxy penetrates through the connected micro-and macro-pores to help estimating mainly the porosity, whereas the Alizarin-Red-S solution is used to identify the cement types, e.g. dolomite and calcite [42]. Many images were captured using a microscope at different magnifications of 2x, 4x, and 10x as illustrated in Fig 4A–4C respectively.

#### 3.2.3 Focused Ion Beam Scanning Electron Microscopy (FIB-SEM)

**Fig 5:**
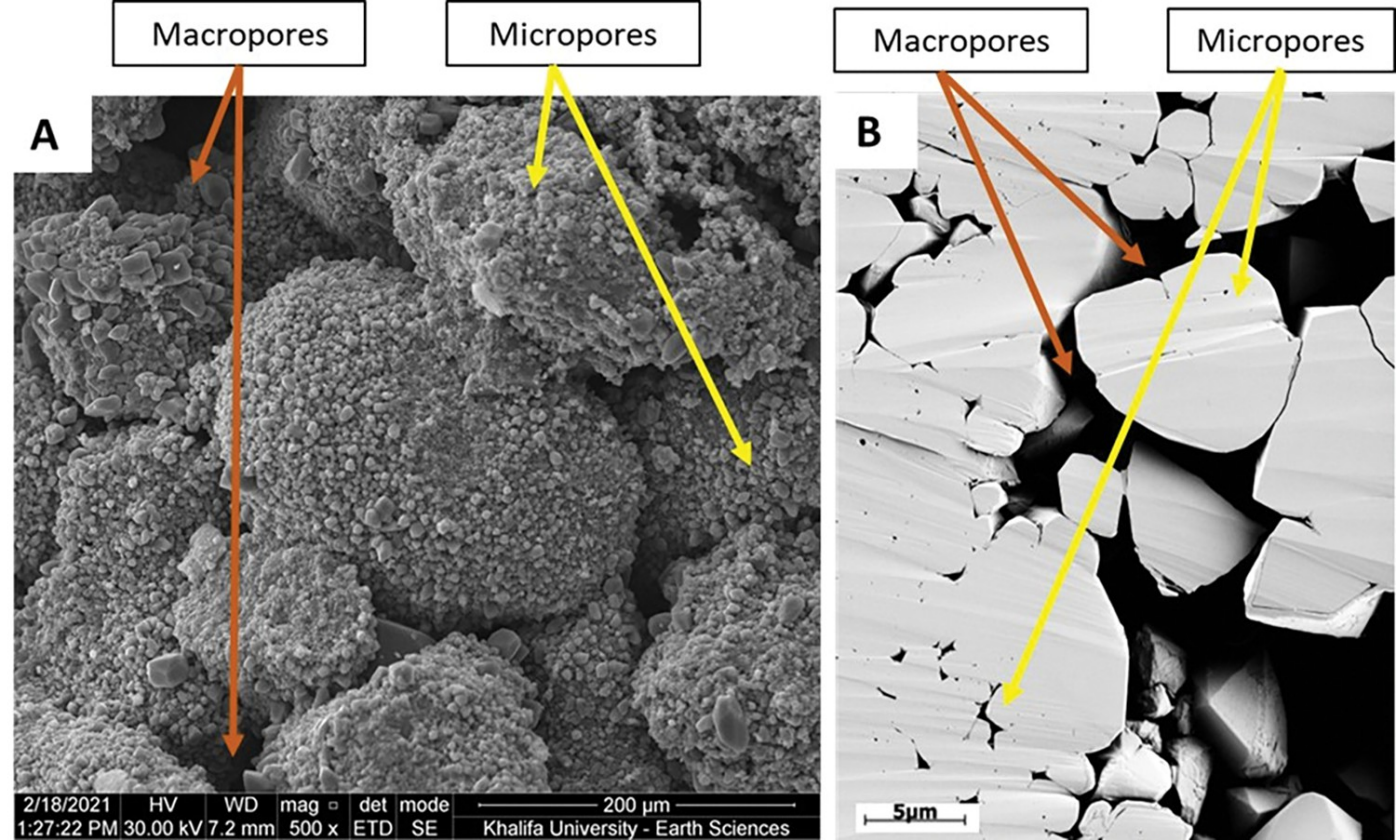
(A) SEM image on freshly broken surface sample showing a 3D view. (B) FIB-SEM image showing a 2D representation. Both images represent the micropores inside the micritized grains (within grain microporosity) and macropores between the peloids (Intergranular pores).

SEM images were acquired on broken surfaces (without polishing or thinning of the rock surface), which then were coated by a thin layer of gold-palladium using Quanta200 instrument. Conventionally, SEM is used to examine the micropores and micro-cements that were not resolved at thin section, as well as to study the changes of the crystal shapes due to the precipitation of macro- and micro-calcite cements. A general observation is the 3D overview of the grains or micrites with variety of grey colors in the sample, which renders it difficult to compute the micro and macro-porosities from the 3D SEM (Fig 5A). Therefore, Focused Ion Beam SEM (FIB-SEM) technique was used on polished surface and coated with conductive material (carbon) without resin injection to prevent any charge and ensure the best imaging quality. This method was applied using FEI scanning electron microscope at Halliburton Ingrain Laboratory in Abu Dhabi. This 2D imaging method ensures a flat surface for a constant relief effect revealing the pores and grains from variable surfaces within the same sample (**Fig 5B**). The images were captured first at 0.6 μm resolution. Then, different sub-samples were selected to acquire energy-selective backscatter (ESB) images at 0.01 μm resolution for the grain-dominated limestone samples and 0.005 μm resolution for the mud-dominated limestone samples (due to grain size differences). The multiscale procedure provides the maximum resolution to study the pore sizes, pores connectivity, grain morphology, and degree of coalescence.

### 3.3 Image analysis

#### 3.3.1 Image segmentation

The process of scanning the image can lead to the presence of undesired noise and artifacts, thereby causing inaccuracies in the detection of the pore network. Hence, image enhancement was employed using image filtering techniques such as mean filter to effectively eliminate noise and enhance edges [43,44]. Image segmentation was applied to classify pixels (2D) and voxels (3D) into two clusters representing solid and pores. In this paper, the image segmentation process was performed on grey level images such as FIB-SEM and MCT in addition to colored images obtained from thin sections. The available grey level images obtained were acquired at high resolution generating bimodal grey level distributions [45]. Therefore, the segmentation was performed automatically using the Otsu method [46]. This method determines the threshold by minimizing the intra-class grey level variance. Subsequently, the segmented image was utilized for identifying the porosity and pore shapes. The petrographic images show the pore space through the blue epoxy filling the pores. Therefore, to extract the pixels corresponding to the pores, the RGB (Red, Green, and Blue) images were transformed into the HSV (hue, saturation, and value) color system. In the HSV representation, the color is represented by the hue (H) component rendering the manual interval selection easy to implement in order to segment the pores from grains. Both grey level and color segmentation approaches were developed in-house using MATLAB codes available as (S1 and S2 Files). The supporting information section includes the user manual for the MATLAB codes utilized in this study (S3 File).

#### 3.3.2 Image multifractal parameters

The heterogeneity property of pore size geometry can be characterized using the multifractal theory on two dimensional images using the singularity spectrum and generalized dimensions [36,44]. For instance, Xie *et al*. (2010) studied pore-scale using fractal and multifractal characteristics throughout digital images of carbonate petroleum reservoirs [33]. The petrophysical outputs (porosity and permeability) are closely associated with the parameters of fractal and multifractal findings. In addition, Jouini *et al*. (2022) assessed carbonates and sandstones heterogeneity using multifractals analysis throughout MCT multi-scale imaging [45]. The results showed that the information dimension (D_1_) and capacity dimension (D_0_) correlated positively with the digital simulations of porosity and permeability values.

The 2D Box-Counting (BC) method was applied to study the self-similarities patterns in 2D segmented images like the FIB-SEM, through dividing them into a grid of boxes. Generally, this method is used to estimate the fractal parameter of Hausdorff dimension [36]. Boxes analysis is used to determine the pore structure complexity and heterogeneity at multi-length scales by zooming the image in and out. In this study, pore geometry was examined as the main pattern of interest in 2D binary images (FIB-SEM). For a specific side length *ε*, the BC estimates the pores that are present in each box. The number of boxes is quantified with the pore phase *N(ε)*. This process was repeated by covering the 2D FIB-SEM with different boxes of descending square side lengths. Then, the fractal dimension was calculated from the regression straight line slope, which reveals the links between *ln(N(ε))* and *ln(1/ε)* (**Table 3**, Equation (8)). For a system, when its geometry was not expressed by a single fractal dimension, a multifractal analysis was used to reveal a range of fractal dimensions that may fully define these geometric systems. Multifractals parameters are calculated by introducing a probability distribution *P*_*k*_ for each *k*^th^ box as shown in **Table 3**, Equation (9). The boxes size *ε* can be linked to *N*_*α*_(*ε*)~ε^−f (α)^, where *α* is the singularity and *f*(*α*) represents the singularity spectrum as presented in **Table 3**, Equations (6) and (7) [47]. Likewise, the generalized dimension (*D*_*q*_), the mass exponent (*τ*_*q*_), the concentration of pore size distribution (*α*_*0*_), the asymmetry degree in the vertical axis *Δf* = *f*(*α*_*min*_)−*f*(*α*_*max*_), and the non-uniformity *Δα* = (*α*_*min*_-*α*_*max*_) are summarized in **Table 3**.

In this study, we implemented multifractal concepts using MICP and FIB-SEM to study different pore types developed in the Arab D limestones. From the integrated data and results, the studied pores in limestones are divided into macroporosity including: intragranular and intergranular, and microporosity including within grain and within matrix micropores, according to their position relative to the micrite particles and grains. Then, pore space geometry and heterogeneity characteristics are discussed based on multifractal theory using data derived from MICP and SEM images.

## 4. Results and discussion

This section focuses on exploring multi-scale imaging methods and image analysis techniques utilized for characterizing the geometry, heterogeneity, and pore connectivity of both macro- and micro-porosities for the six carbonate samples tested. These methods include thin sections, SEM, FIB-SEM, and MCT image analyses. Furthermore, we present the findings of data integration and interpretations, while also addressing the limitations of the employed methods.

### 4.1 Optical microscopy

**Fig 6:**
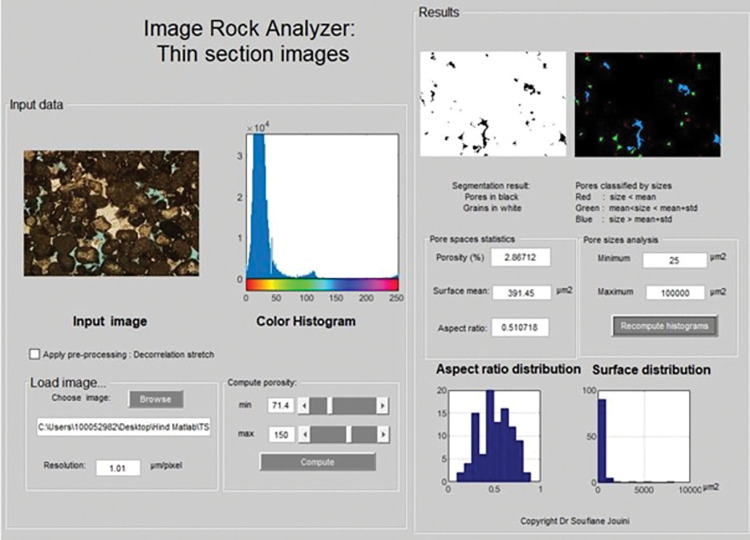
In-house MATLAB Interface for thin section image segmentation. The results obtained include porosity, the distribution of surface area, and the distribution of aspect ratio of sample 1G.

Using an optical microscope, impregnating thin sections with blue epoxy simplifies the pore quantifications step as the resin is penetrating the pore spaces. However, the pore spaces in mud-dominated limestone samples could not be observed due to the small size and highly coalesces micrite. For this reason, only one sample from mud-dominated limestones with relatively high porosity was examined. The quantification of macro-and microporosity in the thin section analysis was applied at magnifications 2x, 4x, and 10x using our in-house MATLAB codes. Each image was processed using decorrelation stretching algorithm to enhance the image colors with band-band correlations [48]. Pixels corresponding to pores were extracted by manually selecting the range of blue colors. The final result was a binary image where black and white pixels represent pores and grains, respectively (**Fig 6**). The porosity was calculated as the ratio of black pixels by the total number of pixels in the image. In order to better interpret visually the results, the pore spaces were classified according to their sizes as illustrated in **Fig 6**. Pore geometry was characterized by calculating surface area, equivalent radius, and aspect ratio for every pore detected. The Aspect ratio is defined as the ratio of minor axis size to the major axis size (**Fig 6**).

**Fig 7:**
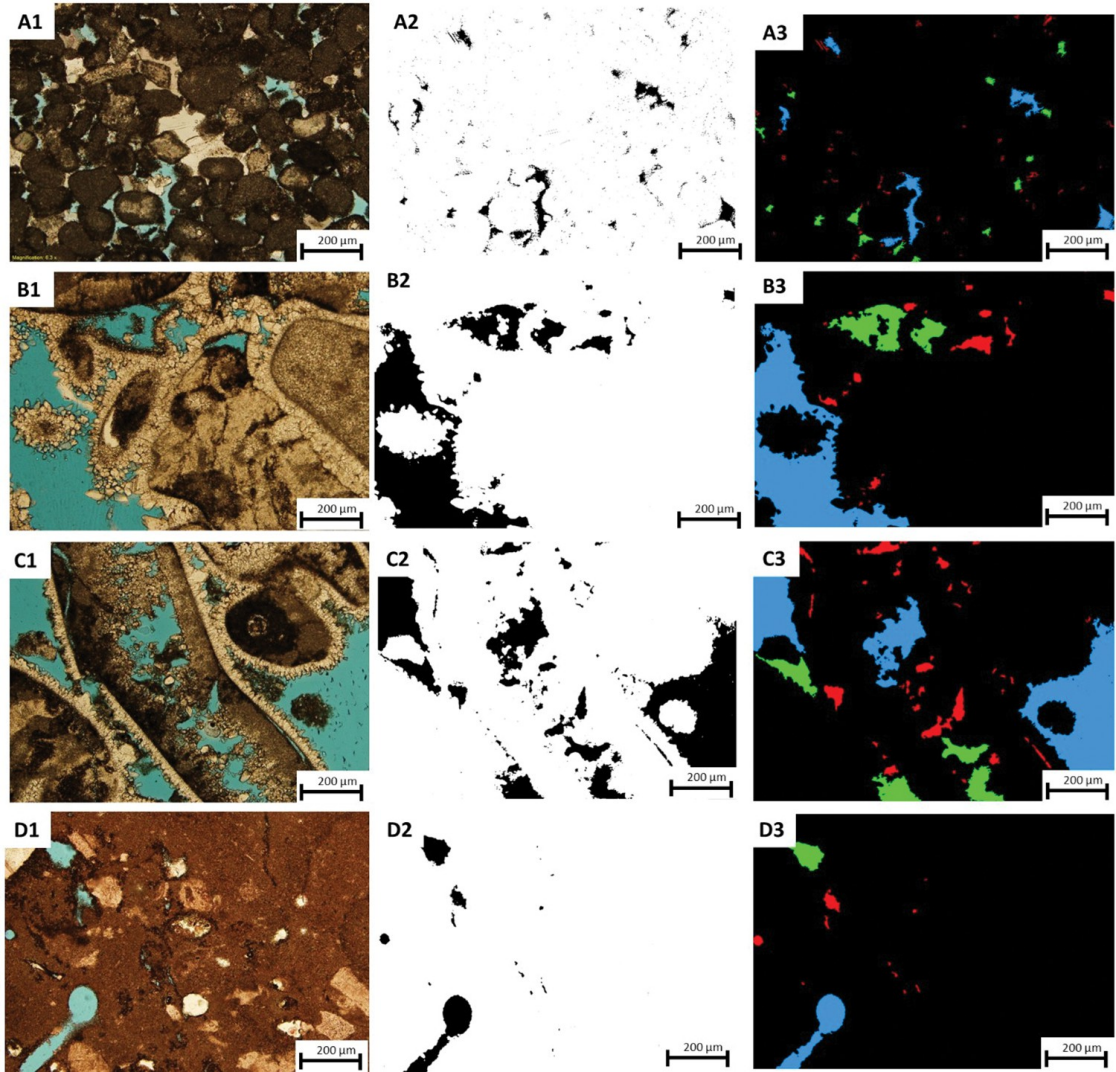
(1) Thin section photomicrographs of the studied samples at 1.01μm resolution of (A) sample 1G, (B) sample 2R, (C) sample 3R, (D) sample 5W. (2) The resulting output after segmentation process where the black colors showing the pores and white colors display grains. (3) Segmented pore spaces are presented in three colors of red, green, and blue based on the different pore sizes ranges.

In general, a disparity between the gas and image porosity values is expected due to the restricted image acquisition area of 2.5 mm by 3.4 mm and 1mm by 1.3 mm at 4x and 10x magnifications, respectively. The captured area is not representative of the entire sample but allows capturing pore geometries at that scale. In addition, micropores smaller than 10μm could not be observed and detected from thin sections. Sample 1G (peloidal grainstone) was analyzed with magnification of 10x (1.01μm resolution) due to the limitation of the intergranular and intragranular porosity that resulted from the dissolution process (**Fig 7, A1-3**). The porosity percentage obtained through image segmentation is 2.87%, but it significantly differs from the helium and MICP porosity values of 12.9% and 10%, respectively (**Table 2**) confirming our assumption mentioned above.

Sample 2R (rudstone) underwent analysis at 2x, 4x, and 10x magnifications, resulting in porosity values of 5.51%, 10.06%, and 14.75%, respectively (S2 Fig and **Fig 7, B1-3**). These varying porosity values are attributed to the different magnifications and views used to capture the sample. Notably, the porosity detected from thin section increases with higher magnification. The porosity values obtained at 4x and 10x closely align with the gas porosity (14.6%) and MICP porosity (13%), indicating that the pores observed in the TS are representative of the macropores in sample 2R. Similarly, sample 3R (rudstone) was subjected to 2x, 4x, and 10x analyses, yielding porosity values of 10.48%, 20.43%, and 19.86%, respectively (**Fig 7, C1-3**). The porosities observed at 4x and 10x are in agreement with each other, suggesting that the imaged area adequately represents the pore spaces measured by gas porosity (17.6%). On the other hand, sample 5W, representing a wackestone texture, exhibited relatively low helium porosity (6.8%). The sample was analyzed at a high magnification of 10x to capture the maximum porosity (2.6%) (**Fig 7, D1-3**). This outcome could be attributed to the lack of representativeness in the thin section. In **Table 4**, the results for porosity quantification, surface area, and aspect ratio averages based on TS analysis for the four samples is summarized. These parameters offer a quantitative geometric description of pore sizes and prove to be advantageous in providing valuable insights.

**Table 4:** Quantifications of porosity from the TS along with surface area mean and aspect ratio at magnification 10x, for corresponding helium porosity values see Table 2.

Sample	Porosity (%)	Surface Area Mean (μm^2^)	Aspect Ratio Mean
1G	2.87	391.45	0.51
2R	14.76	6889.54	0.58
3R	19.86	5485.85	0.50
5W	2.61	2931.26	0.51

### 4.2 FIB-SEM

**Fig 8:**
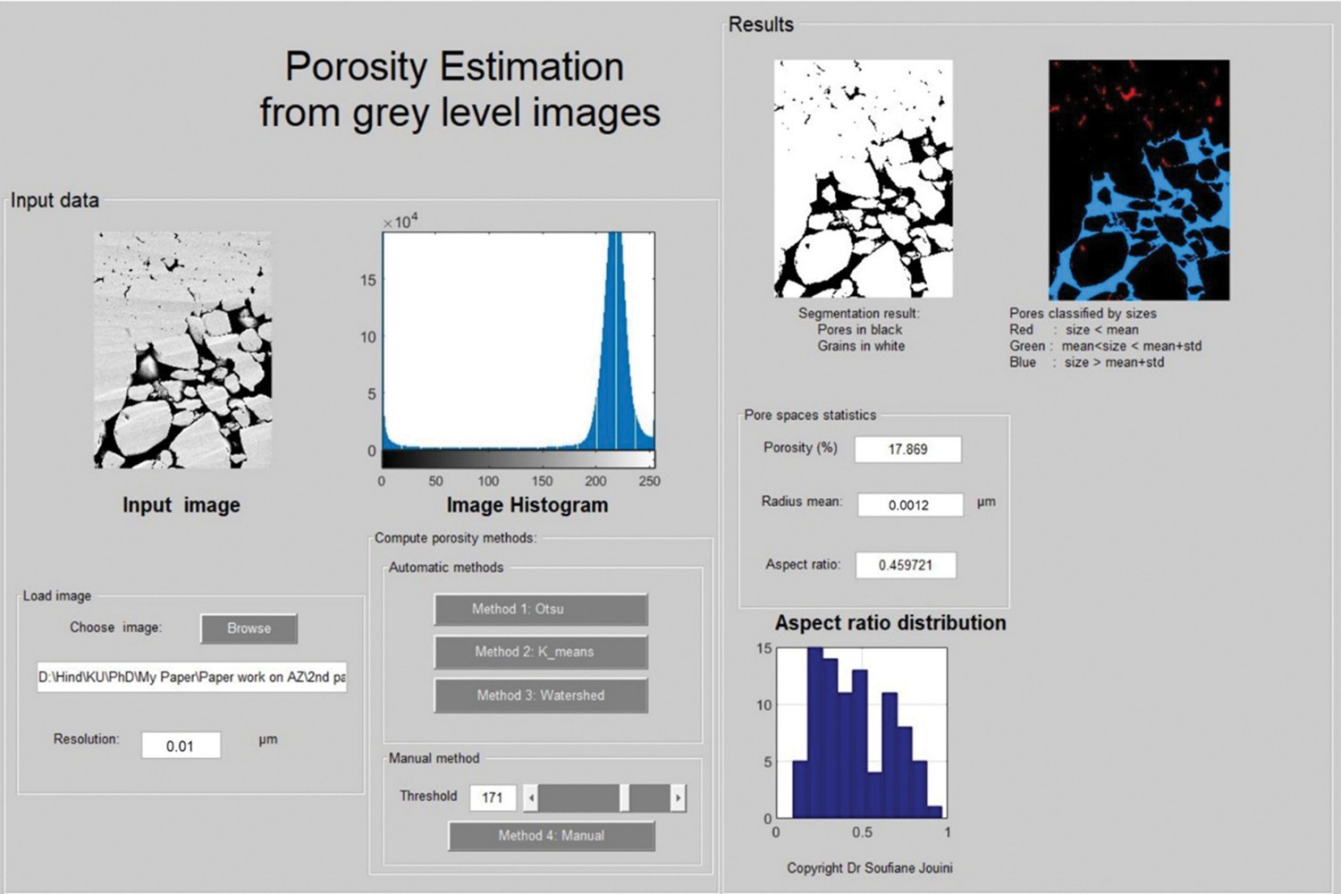
Porosity and pore structure characterization from FIB-SEM images using Otsu’s method as thresholding segmentation technique.

The quantification of macro-and micro-porosity on focused ion beam SEM images (FIB-SEM) was achieved by segmentation using Otsu’s method (**Fig 8**). The FIB-SEM image is made of grey scale intensities between 0 and 255. The high grey levels are showing the grains and low grey scale ones are revealing the pores. The quantification was implemented at 0.01 μm resolution for the grain-dominated limestones and 0.005 μm for the mud-dominated limestones (due to the pore size). Several geometrical parameters were calculated from the segmented images such as surface area, equivalent radius, and the aspect ratio of each pore. The total porosity was obtained as the ratio of the total number of pores identified as pixels to the total number of pixels. The interface was developed in-house using MATLAB (**Fig 8**).

**Fig 9:**
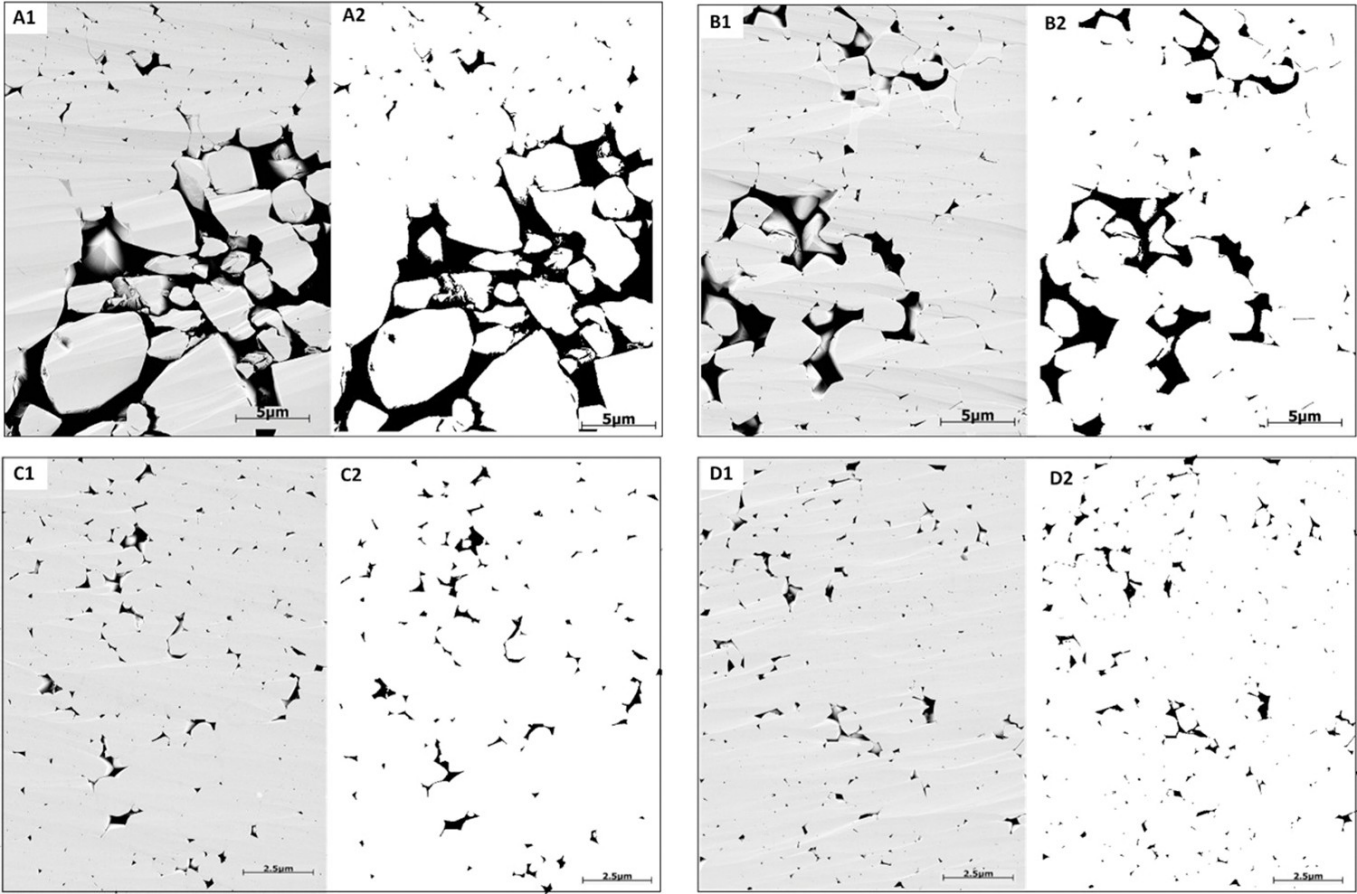
(1) Original and (2) segmented FIB-SEM images where the black colors show the pores and white colors reveal grains. FIB-SEM images of the studied samples. (A) sample 1G, (B) sample 2R, (C) sample 4M, and (D) sample 5W.

Sample 1G (peloidal grainstone) underwent analysis using FIB-SEM imaging at a resolution of 0.01 μm to capture and quantify various pore spaces, including intergranular and intragranular porosity resulting from dissolution, as well as part of the microporous system within the micritized grains (**S1 Fig**). The porosity calculated from image segmentation is 17.9% (**Table 5**), which exceeds the gas porosity (12.9%) (**Fig 9A**). This discrepancy can be attributed to the limitation in the field of view (FOV) as the area captured by the FIB-SEM image is approximately 23μm by 31μm (0.01 μm resolution). The FIB-SEM results are more consistent with the gas porosity compared to thin section segmentation, primarily due to the optical microscope’s inability to detect micropores in the thin section. Similarly, sample 2R (rudstone) was analyzed using images acquired at a resolution of 0.01 μm to ensure the inclusion of all pore types, such as intergranular macropores and within grain microporosity (inside the micritized peloids) (**Fig 9B**). The resulting porosity from the FIB-SEM image is 9.31% (**Table 5**), which is lower than both the thin section and gas porosity values (around 14%). This difference might be attributed to the limitation in capturing macropores in FIB-SEM images (23μm x 31μm) compared to thin section images (1mm x 1.3 mm) at 10x magnification. Additionally, the mud-supported samples were analyzed using images acquired at a resolution of 0.005 μm due to the small pore size. The segmented images reveal that pores primarily occur between the micrite particles (matrix microporosity) (**Fig 9C and 9D**). The mud-dominated samples (4M, 5W, and 6M) exhibit a microporous system, characterized by geometric parameters like surface area, equivalent radius, and aspect ratio. **Table 5** indicates that the equivalent radius mean for the three mud-supported samples ranges in [9.56 10^−4^, 1.12 10^−3^] in μm, whereas the equivalent radius means for the grain-supported samples are larger, exceeding 1.96 10^−2^ μm. Furthermore, the aspect ratio means for all samples fall within the range of [0.41, 0.53].

**Table 5:** Quantifications of porosity from the FIB-SEM along with surface mean and aspect ratio at scale of 0.01 μm to the grain-dominated samples and 0.005 μm for mud-dominated samples using threshold value of 170 for all samples. For helium porosity values refer to Table 2.

Sample	Resolution (μm)	Porosity (%)	Surface Mean (μm^2^)	Equivalent Radius Mean (μm)	Aspect Ratio Mean
1G	0.01	17.9	1.20 x10^-3^	1.96 x10^-2^	0.46
2R	0.01	9.31	1.40 x10^-3^	2.11 x10^-2^	0.41
4M	0.005	2.48	3.94 x10^-6^	1.12 x10^-3^	0.50
5W	0.005	2.61	3.59 x10^-6^	1.07 x10^-3^	0.53
6M	0.005	2.90	2.87 x10^-6^	9.56 x10^-4^	0.46

### 4.3 3D X-ray CT and MCT

**Fig 10:**
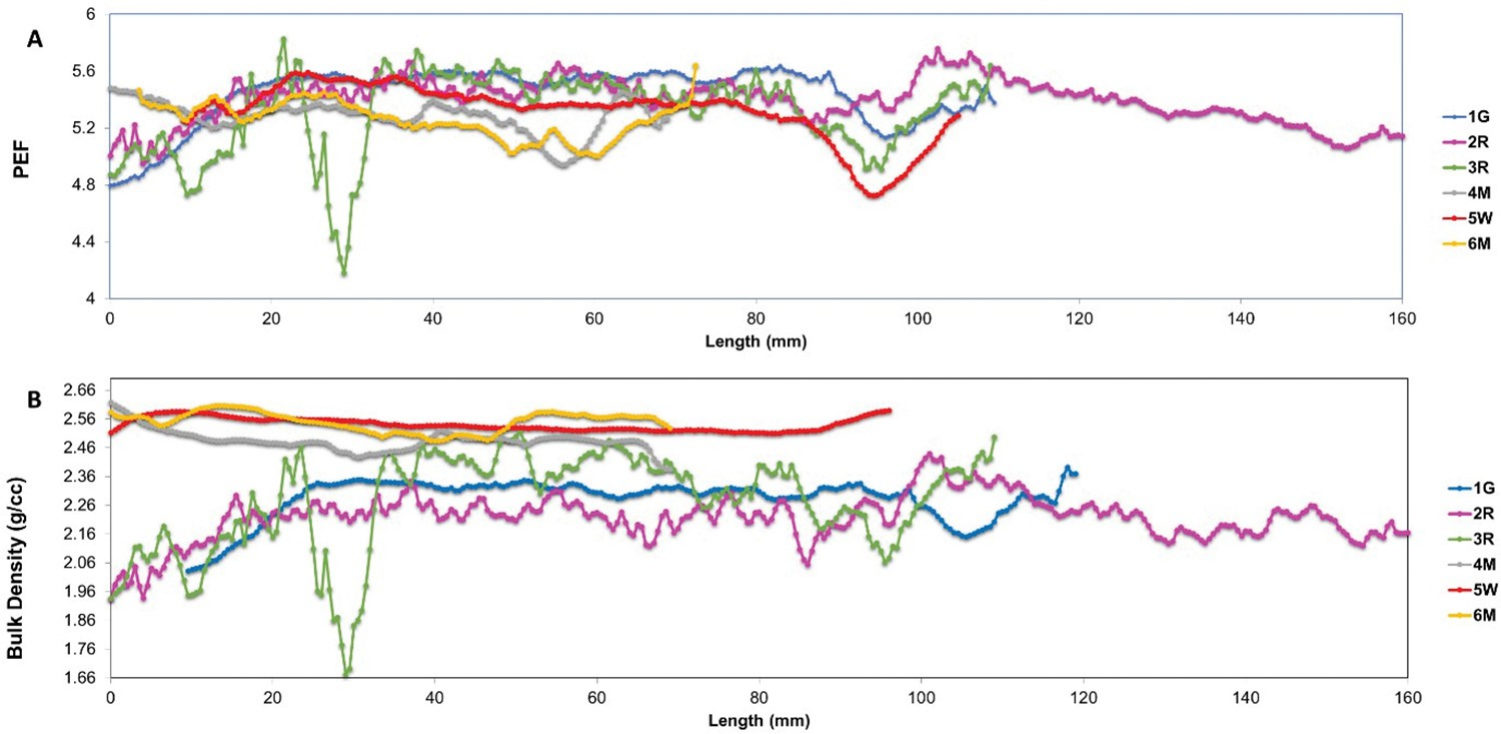
CT-numbers plots of (A) photoelectric factors and (B) bulk density on the studied samples.

**Fig 11:**
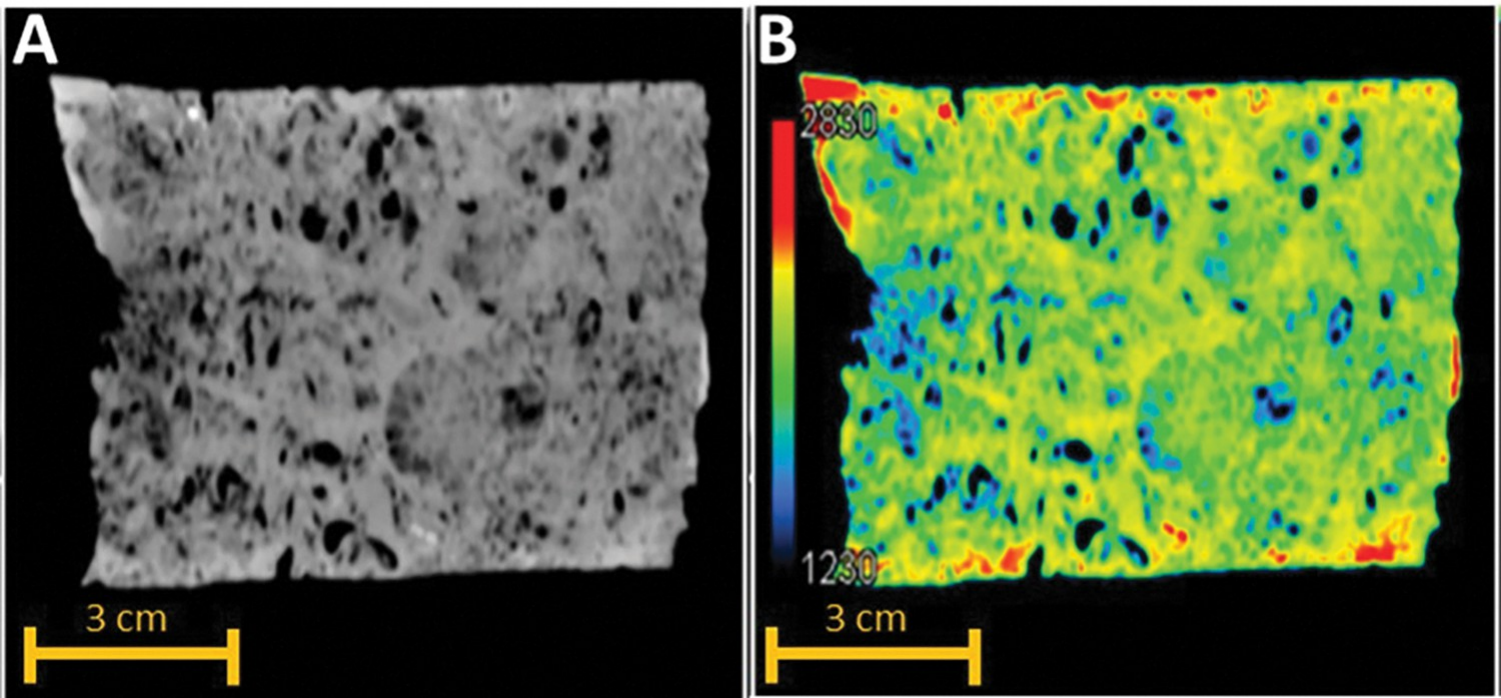
2D vertical cross section from 3D CT-scan of sample 3R. (A) original grey level image, (B) original image illustrated using jet color bar.

The CT scans of grain-dominated limestones samples show general structural heterogeneity. All the carbonate samples show scatter calcite cements occluding the pore spaces. Based on the CT scans, Sample 1G shows abundant micritized peloids. On the other hand, samples 2R and 3R exhibit the most heterogeneous pore geometries among the carbonate samples with grains larger than 2 mm along with visual intergranular porosity. The primary benefits of the CT-scan are to provide insights to the mineralogy using PEF as indicator and the variations of porosity along the core plug samples based on the bulk density parameter. In addition, CT-scan provides a precise understanding of the location from which a sub-plug can be obtained for MCT scanning, MICP, and FIB-SEM techniques. This enables more comprehensive analysis of the pore network within the studied limestones. The samples exhibit PEF values ranging from 4.2 to 5.6, with an average of 5, indicating that calcite cements are the predominant component [38,39] in the studied samples (**Fig 10A**). The drop that happens at particular value of 4.2 for sample 3R, is due to the presence of large vugs at around a height of 3 cm in the sample (**Fig 11**).

The bulk density provides insights on the sample’s porosity, where the low bulk density readings correspond to high porosity values. The bulk density in the grain-supported samples varies between 1.67 to 2.52 g/cc, whereas it ranges between 2.38 to 2.62 g/cc in mud-supported limestones samples (**Fig 10B**). This confirms that the grainy samples have higher porosity than muddy samples with higher bulk density values.

**Fig 12:**
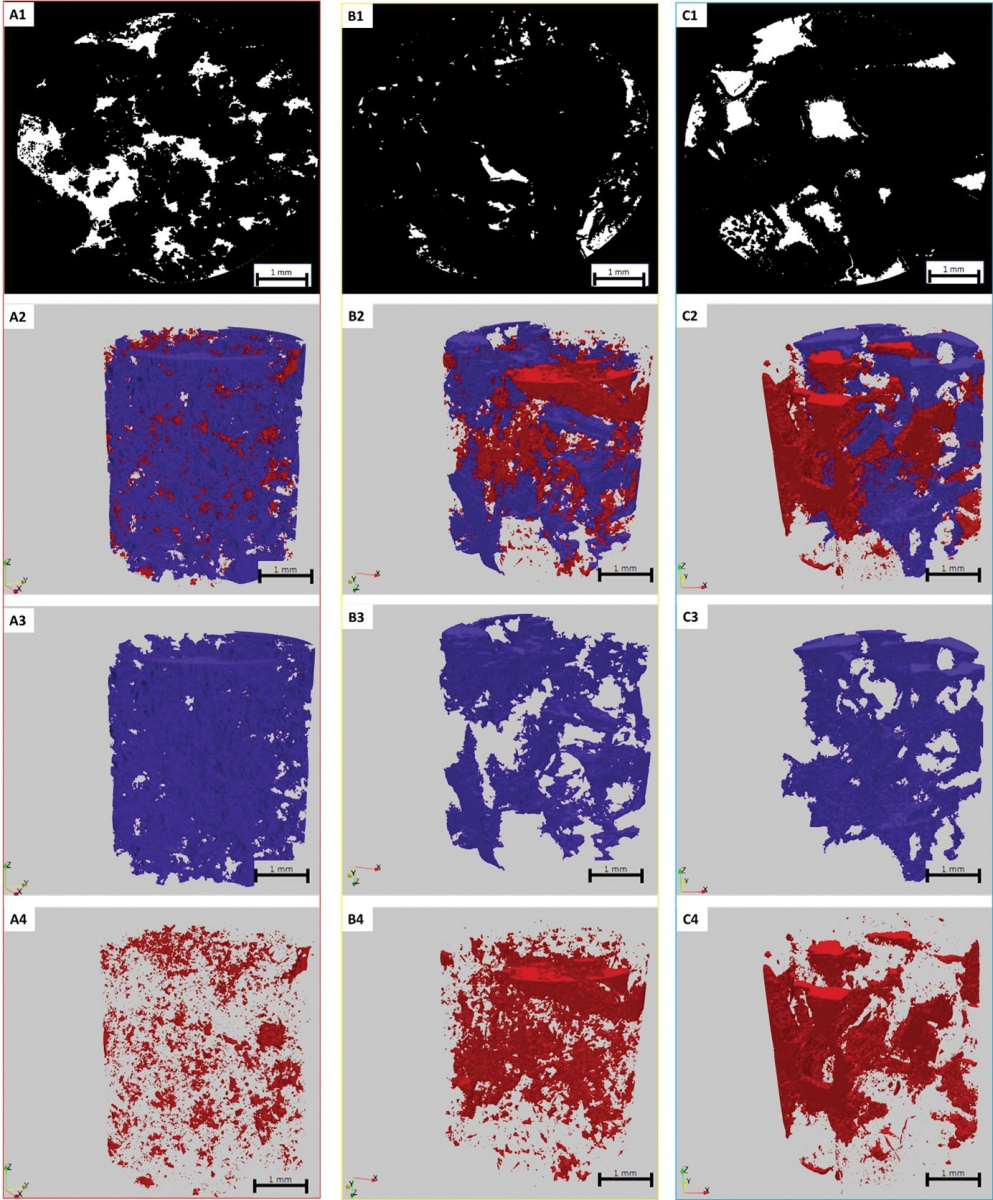
Digital image processing results of the three studied samples of grain-dominated limestones with diameter subset 4 mm using MCT. (A1-C1) after segmentation, (A2-C2) connected and isolated pores, (A3-C3) connected pores, (A4-C4) isolated pores. The blue color represents connected pores and red color represents isolated pores.

All sub-plugs with a diameter subset of 4 mm underwent MCT scanning at a resolution of 4 μm. To reduce the noise resulting from the acquisition process and extract the pore network, the images were filtered using a 3D mean filter and then segmented using Otsu’s algorithm. The connected component algorithm, implemented through ParaView Software (**Fig 12**), was utilized to distinguish and classify connected pores from those that were isolated in the 3D images. The percentage of calculated connected and isolated pores for the three grain-dominated samples is presented in **Table 6**. Among these samples, the peloidal grainstones (1G) exhibited the largest connected pore volume compared to the other two samples (**Fig 12A**). However, the digital results do not align with the experimental results of helium porosity and MICP. This discrepancy could be attributed to the limited volume of sub-samples scanned, which were relatively small, around 64 mm^3^ only. Additionally, it is important to note that the pore network estimation using digital images was obtained under dry sample conditions, and the image resolution of 4μm might not capture most of the pore throats, leading to the detection of isolated pores in the images that are connected in reality. This limitation should be considered when interpreting the results from this approach.

**Table 6:** DRP results after MCT-scans segmentation and connectivity analysis.

Samples	Connected Pore (%)	Isolated Pore (%)
1G	94.6	5.4
2R	57	43
3R	59	41

### 4.4 Multifractal analysis

**Fig 13:**
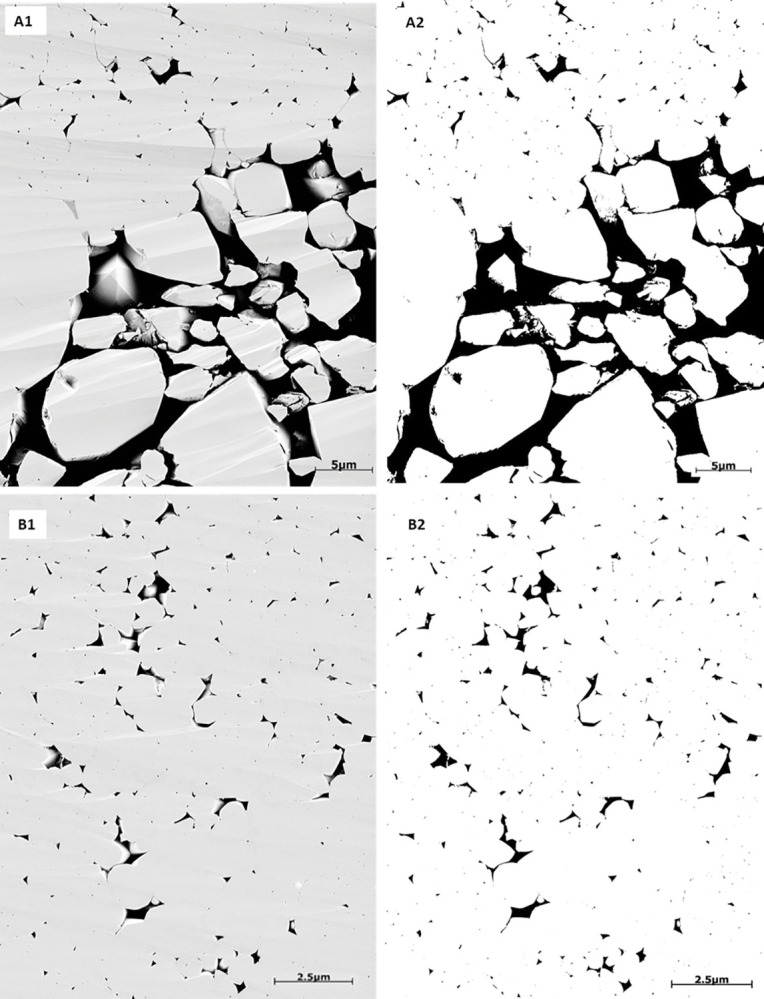
Image segmentation for samples: (A) sample 1G, and (B) sample 4M. (A1-B1) original images. (A2-B2): binarized image results, where the black pixels denote pores and white pixels show grains.

This section utilized multifractal analysis to assess the heterogeneity of pore geometries at two distinct length scales, using data from FIB-SEM images and MICP experimental dataset. In FIB-SEM images, grains and pores are represented in light gray and black, respectively (**Fig 13, A1-B1**). Throughout this study, pores were segmented from FIB-SEM images using Otsu’s thresholding method and are represented in black colors (**Fig 13, A2-B2**). Our analysis was obtained at FIB-SEM image scale of 23μm by 31μm (0.01 μm resolution) for grain-dominated limestones and 11 μm by 15 μm (0.005 μm resolution) for the mud-dominated samples. Intergranular pores are irregular shape due to the precipitation of calcite spars between the grains, and their abundance is quite common over a wide range with an uneven distribution based on FIB-SEM observation (**Fig 13**). In addition, within grain microporosity is also largely observed in the studied samples within the micritized peloids due to micro-organisms activities [4,49], with pore size less than 10 μm (**Fig 13**). However, in mud-dominated samples, FIM-SEM shows abundant matrix microporosity that are originally formed during deposition but were modified due to the diagenetic alterations. This is noted from the change in micrites morphology (size and shape), which can lead to different pore geometries and pore throat sizes [7,10,50].

**Fig 14:**
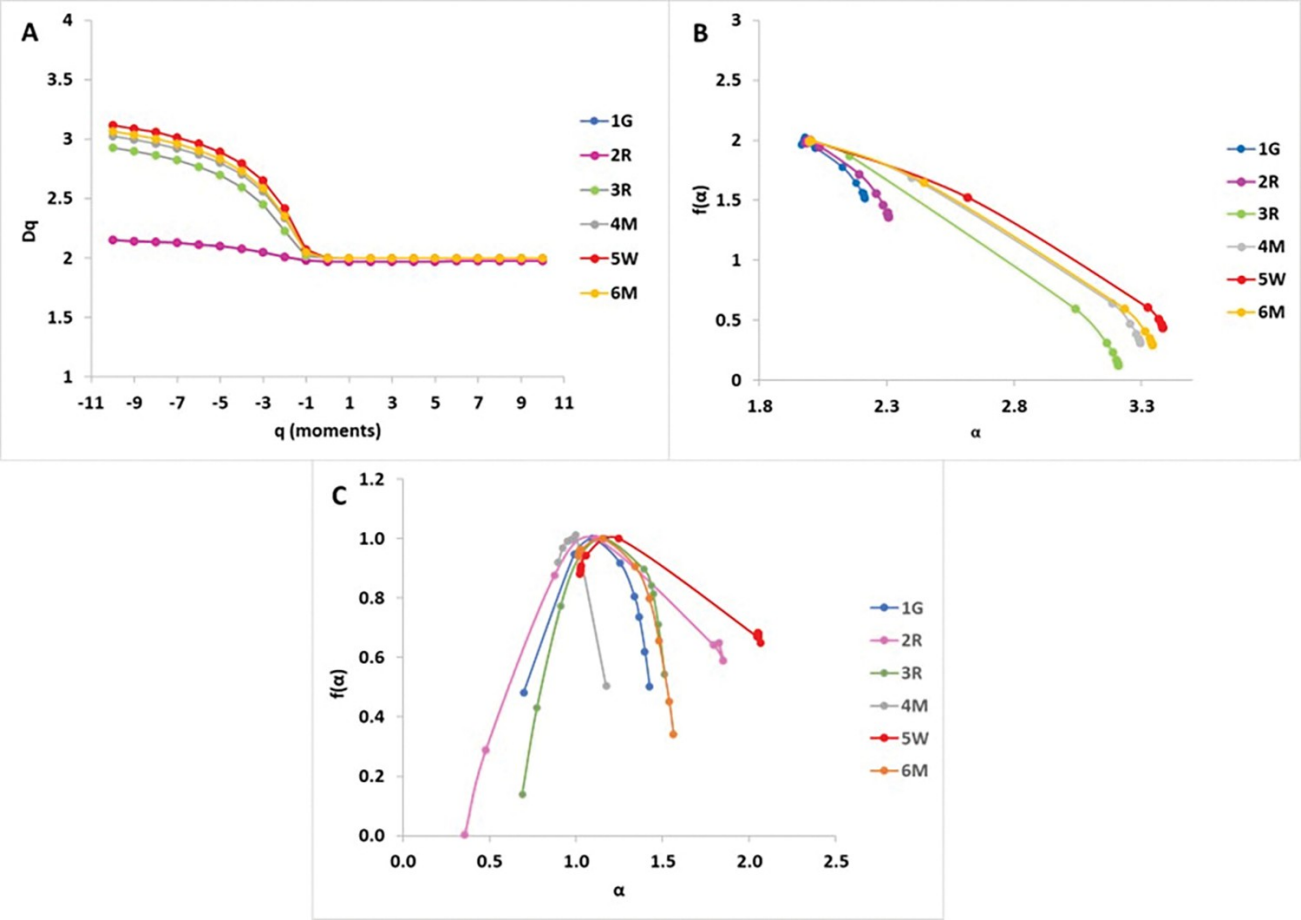
(A) Multifractal dimensions (D_q_) associated to the FIB-SEM image of the studied samples along with moments order (q) between −11 and11. (B-C) multifractal spectrum of spatial distribution of different pore extraction images for the studied samples. (B) from FIB-SEM imaging; (C) from MICP analysis.

The multifractal dimensions (D_0_), non-uniformity degree (Δα), and the asymmetry degree in the vertical axis Δf(α) values from FIB-SEM were reported in **Table 7. Fig 14A** illustrates the multifractal dimensions with respect to the moments and **Fig 14B** displays singularity spectrum curves for the six samples. In most of the samples (1G, 3R, 4M, 5W, and 6M), the generalized dimension D_q_ showed a relatively high rate decrease leading to a convergence toward a constant value for moments q ≥ -1 (**Fig 14A**) reflecting the high heterogeneity of the samples. Nevertheless, in sample 2R, the generalized dimension D_q_ shows a slow rate decrease leading to a convergence toward a constant value for moments q ≥ -1, as illustrated in **Fig 14A**. The above observations support that D_q_ helps in determining the samples heterogeneity degree, since their values would drop to a constant faster in images where pore distributions were less heterogeneous when comparing the mud-dominated and grain-dominated limestones separately (**Fig 14**). In addition, the heterogeneous multifractal distributions have a wide concave f(α)-spectra as opposed to narrow concave f(α)-spectra for the homogeneous samples [51,52].

**Table 7:** Estimations of singular exponents and multifractal spectrums values from MICP and FIB-SEM analyses.

Samples No.	From FIB-SEM			From MICP		
	D_0_	Δα	Δf(α)	α_0_	Δα	Δf(α)
1G	1.97	0.25	0.51	1.09	0.73	0.52
2R	1.98	0.32	0.64	1.11	1.49	0.99
3R	1.99	1.21	1.87	1.16	0.82	1.01
4M	1.99	1.30	1.96	1.01	0.51	0.28
5W	1.99	1.39	1.56	1.25	1.04	0.35
6M	1.99	1.35	1.70	1.15	0.55	0.66

The dataset illustrates the singularity f(α) for six samples, displaying right-sided asymmetric convex parabolic curves (**Fig 14B**). **Table 7** summarizes the multifractal parameters derived from the FIB-SEM images for these samples. The fractal dimension (D_0_) values range from 1.96 to 1.99 for the studied images. Among the samples, 3R, 4M, 5W, and 6M exhibit the highest Δα values, falling within the range of [1.21, 1.39] (**Table 7**), indicating that these samples are the most heterogeneous.

For instance, in sample 4M, the FIB-SEM image analysis (**Fig 13B**) reveals various sizes of segmented micropores within the image area of 11 μm by 15 μm (0.005 μm resolution), resulting in a large Δα value. On the other hand, samples 1G and 2R have Δα values of 0.25 and 0.32, respectively (**Table 7**). In sample 1G, the FIB-SEM analysis (**Fig 13A**) demonstrates abundant large segmented macropores with equivalent sizes, leading to a homogeneous segmented image. This observation is further supported by the relatively low values of Δα and Δf(α), which are 0.25 and 0.5, respectively (**Table 7**). These results confirm that Δα and Δf(α) effectively capture complexity and heterogeneity in FIB-SEM images.

**Fig 15:**
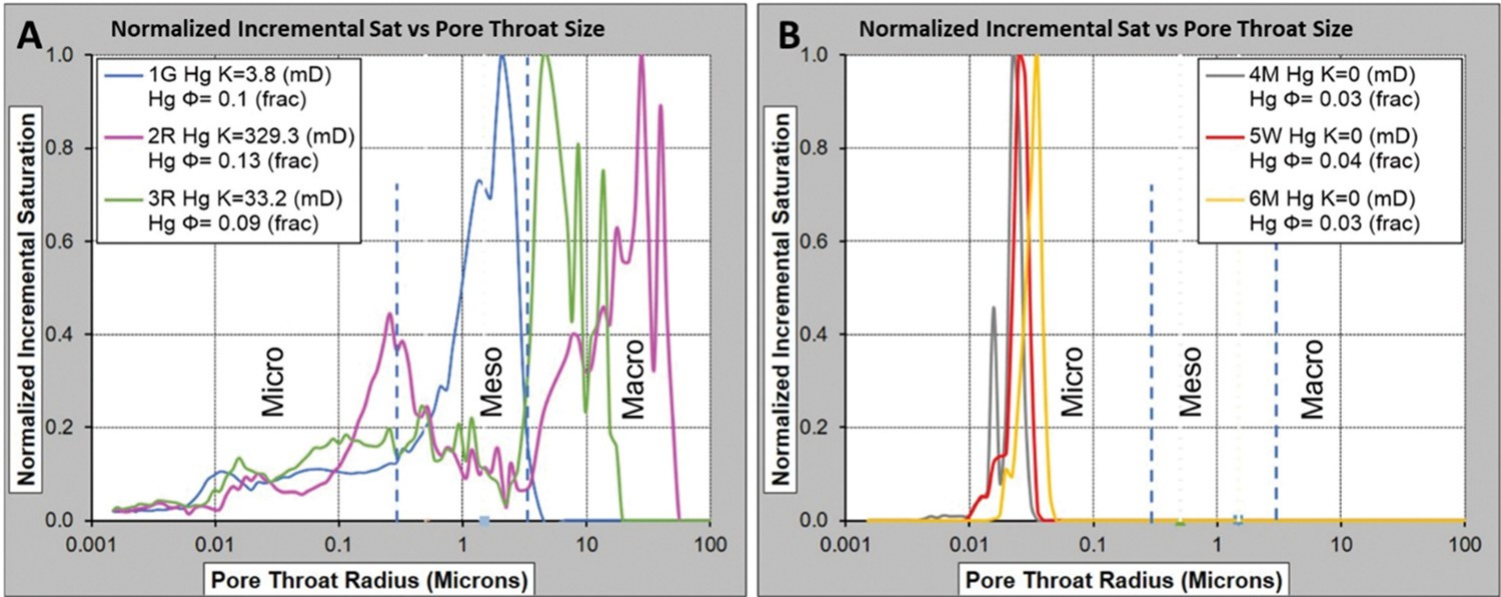
Pore-throat size distribution calculated from the MICP curves for (A) grain-dominated and (B) mud-dominated limestones.

The MICP analysis shows that mud-supported samples are associated to uni-modal pore throat system, which is most probably micropores with a size range between 0.02–0.06 μm, while the grain-supported limestones are associated to dual-pore throats systems (micro: 0.01–0.03 μm -and meso-to-macropores: 2–70 μm) (**Fig 15**). The smaller and tighter the pores and pore throats need higher amount of injected mercury than in the more porous grain-dominated limestone samples. The results obtained from the MICP analysis for sample 1G validate our findings from the thin section analysis, which also revealed a discrepancy in porosity values. The MICP curve (**Fig 15A**) clearly indicates that a majority of the pore throat sizes are less than 5 μm. Furthermore, the MICP analysis for sample 2R demonstrates that a significant portion of the pore throat sizes is greater than 5 μm, which corroborates the consistency between the TS analysis and the experimental porosity results obtained through gas and MICP techniques. Apart from sample 3R, **Table 2** indicates a satisfactory correlation between the gas and MICP permeability values for all samples. The divergence in permeability and porosity values in sample 3R can be attributed to two possible factors: (i) The heterogeneity in pore throat geometry, as illustrated in **Fig 15A**, (ii) The inadequate representativeness (due to sampling) of the trim compared to the core plug sample. The rock properties obtained from gas and MICP experiments for the mud-dominated limestones (4M, 5W, and 6M) display good consistency. This agreement can be attributed to the relative homogeneity of the samples, which display a dominant microporous system (**Fig 15B**).

The singularity spectrum graphs of the six samples (**Fig 14C**) exhibit convex parabolic curves, indicating multifractal behaviours in the pore distributions. To investigate the heterogeneity and complexity characteristics of the rocks, singularity parameters Δα, Δf(α), and pore size concentration α_0_ were assessed (**Table 7**). The α_0_ values for all six samples fall within the range of [1.01, 1.17]. Additionally, positive Δf(α) values across all samples suggest that a dominant probability subset is present. Samples 4M and 6M show the lowest Δα values of 0.51 and 0.55, respectively, indicating that these samples are the most homogeneous (**Fig 14C**). This observation is supported by their low Δf(α) values of 0.28 and 0.66, respectively. On the other hand, samples 1G, 2R, 3R, and 5W exhibit higher pore structure distribution heterogeneity, with Δα values ranging from 0.73 to 1.49, and Δf(α) values ranging from 0.35 to 1.01 (**Table 7**). Furthermore, the singularity spectra curves f(α) for samples 4M, 5W, and 6M display right-sided asymmetry around α equal to 1 for 4M and 1.3 for samples 5W and 6M (**Fig 14C**). In contrast, samples 2R and 3R exhibit left-sided asymmetry. The singularity curves of samples 4M, 5W, and 6M reveal wider right portions with sharper slopes compared to the left ones.

The findings and observations validate that multifractal parameters Δα and Δf(α) are effective for quantifying and characterizing rock heterogeneity. While the primary objective of this study was to independently examine pore structures across various length scales, we noted a consistent characterization of heterogeneity using multifractals in both the MICP (approximately 2.5 cm scale) and FIB-SEM (approximately 25 μm scale) analyzed samples. This observation supports the potential relationship between the scales employed in the sample analysis. Finally, the main limitation of the proposed approach is the absence of reference values of Δα and Δf(α) in the literature, which would allow for the classification of samples into homogeneous and heterogeneous groups.

## 5. Conclusions

In this paper, multi-scale imaging methods and experimental laboratory measurements were applied to characterize macro- and micro-porosity in six carbonate core plugs from Arab D formation in UAE. The main findings of this work are as follows:

- MICP, porosity, and permeability were experimentally measured using special core analysis on the six samples. The mud-dominated limestones showed unimodal distribution of pore throat revealing quite homogenous samples based on the dominant micropores, whereas the grain-dominated limestones revealed bimodal pore throat distribution indicating more complex and heterogeneous samples due to the different pore types (within grain micropores and intergranular macropores).
- Digital multifractal analysis was implemented to investigate the carbonate samples heterogeneity using FIB-SEM images. At this length scale, samples 3R, 4M, 5W, and 6M display the highest Δα values, falling within the range of [1.21, 1.39], showing that these samples are the most heterogeneous. However, 1G and 2R reveal Δα values of 0.25 and 0.32, respectively demonstrating homogeneous samples.
- Experimental multifractal analysis was utilized to study the carbonate samples heterogeneity based on MICP. At this length scale, samples 4M and 6M show the lowest Δα values of 0.51 and 0.55, respectively along with low Δf(α) values, demonstrating that these samples are the most homogeneous. Conversely, samples 1G, 2R, 3R, and 5W exhibit more heterogeneous pore structure, with Δα values ranging from 0.73 to 1.49, and Δf(α) values ranging from 0.35 to 1.01.

## Supporting information

S1 Fig A FIB-SEM image representing grainstone sample (1G). (PNG)

S2 Fig A thin section image representing rudstone sample (2R). (PNG)

S1 File A MATLAB software processing grey level FIB-SEM images. (RAR)

S2 File A MATLAB software processing thin section images. (RAR)

S3 File A MATLAB user-manual software for image processing. (PPT)
