# Supplementary figures and images for: Multiscale investigation of pore structure heterogeneity in carbonate rocks using digital imaging and SCAL measurements: A case study from Upper Jurassic limestones, Abu Dhabi, UAE
Source: PLoS One. 2024 Feb 8;19(2):e0295192. doi: 10.1371/journal.pone.0295192 (PMC10852275; doi:10.1371/journal.pone.0295192)

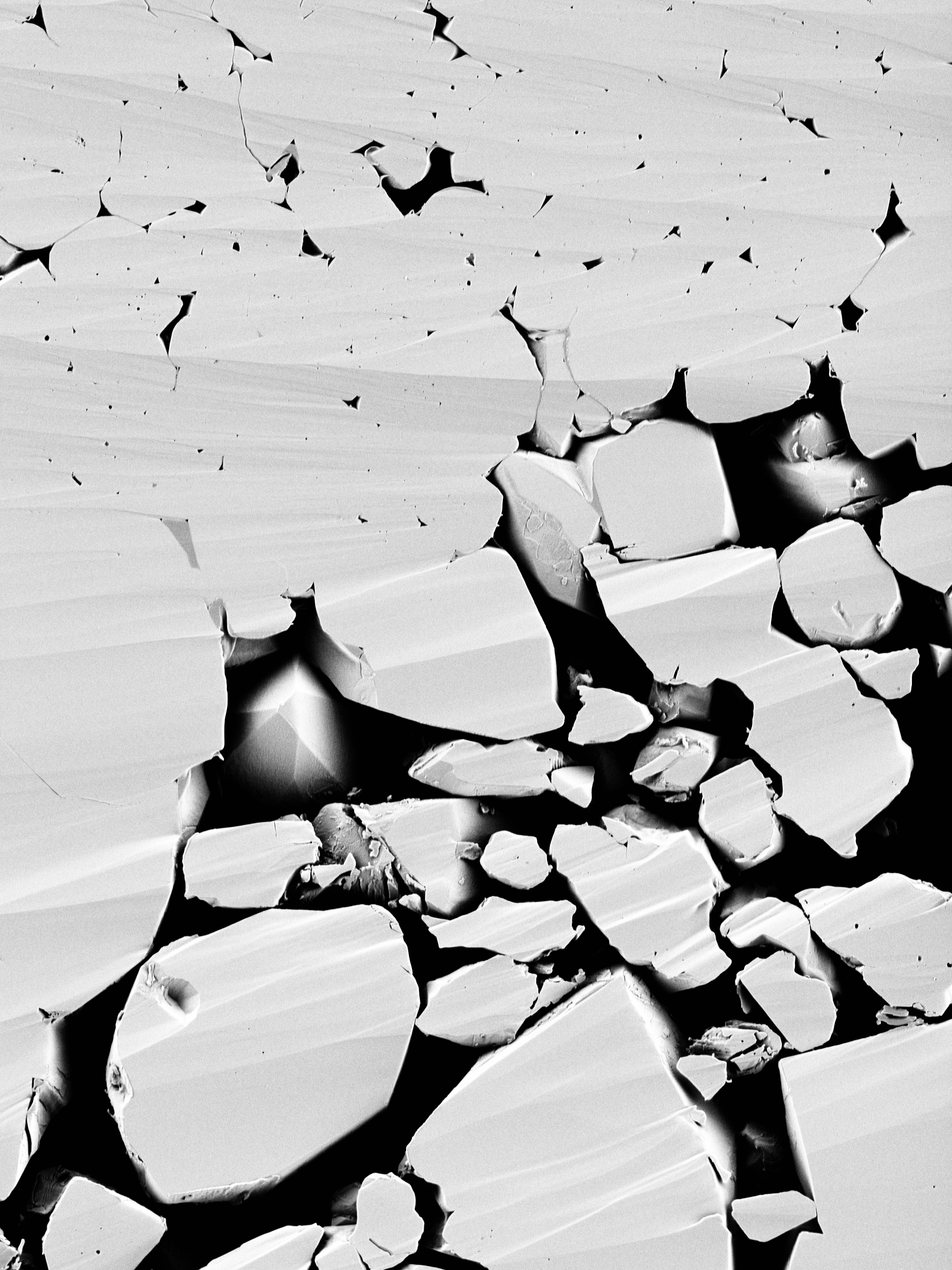

Supplement: S1 Fig — (PNG) [file pone.0295192.s001.png]

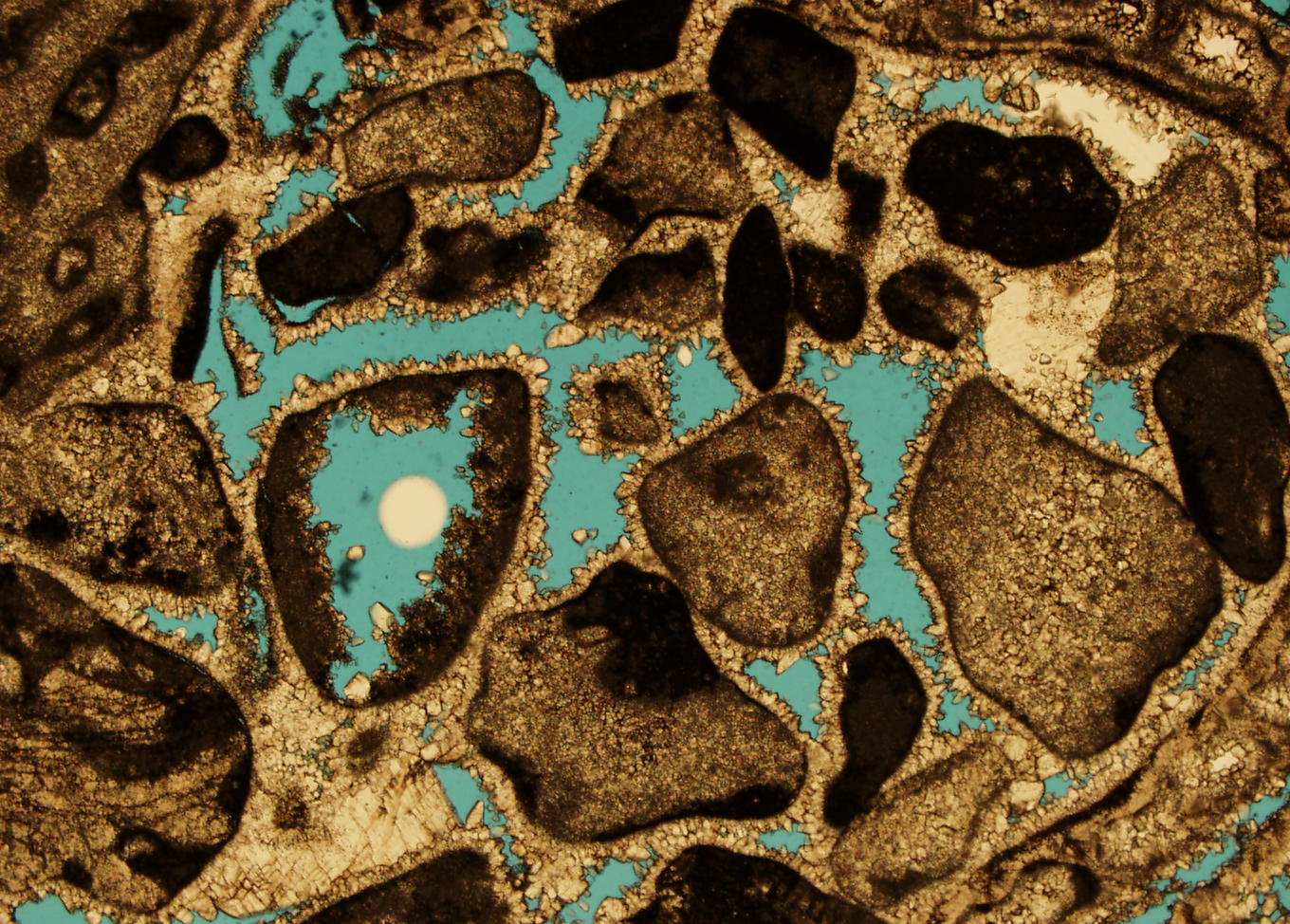

Supplement: S2 Fig — (PNG) [file pone.0295192.s002.png]
