# Supplementary material for: Multiscale investigation of pore structure heterogeneity in carbonate rocks using digital imaging and SCAL measurements: A case study from Upper Jurassic limestones, Abu Dhabi, UAE
Source: PLoS One. 2024 Feb 8;19(2):e0295192. doi: 10.1371/journal.pone.0295192 (PMC10852275; doi:10.1371/journal.pone.0295192)
Supplement: S3 File — (PPT) [file pone.0295192.s005.ppt]

## Slide 1
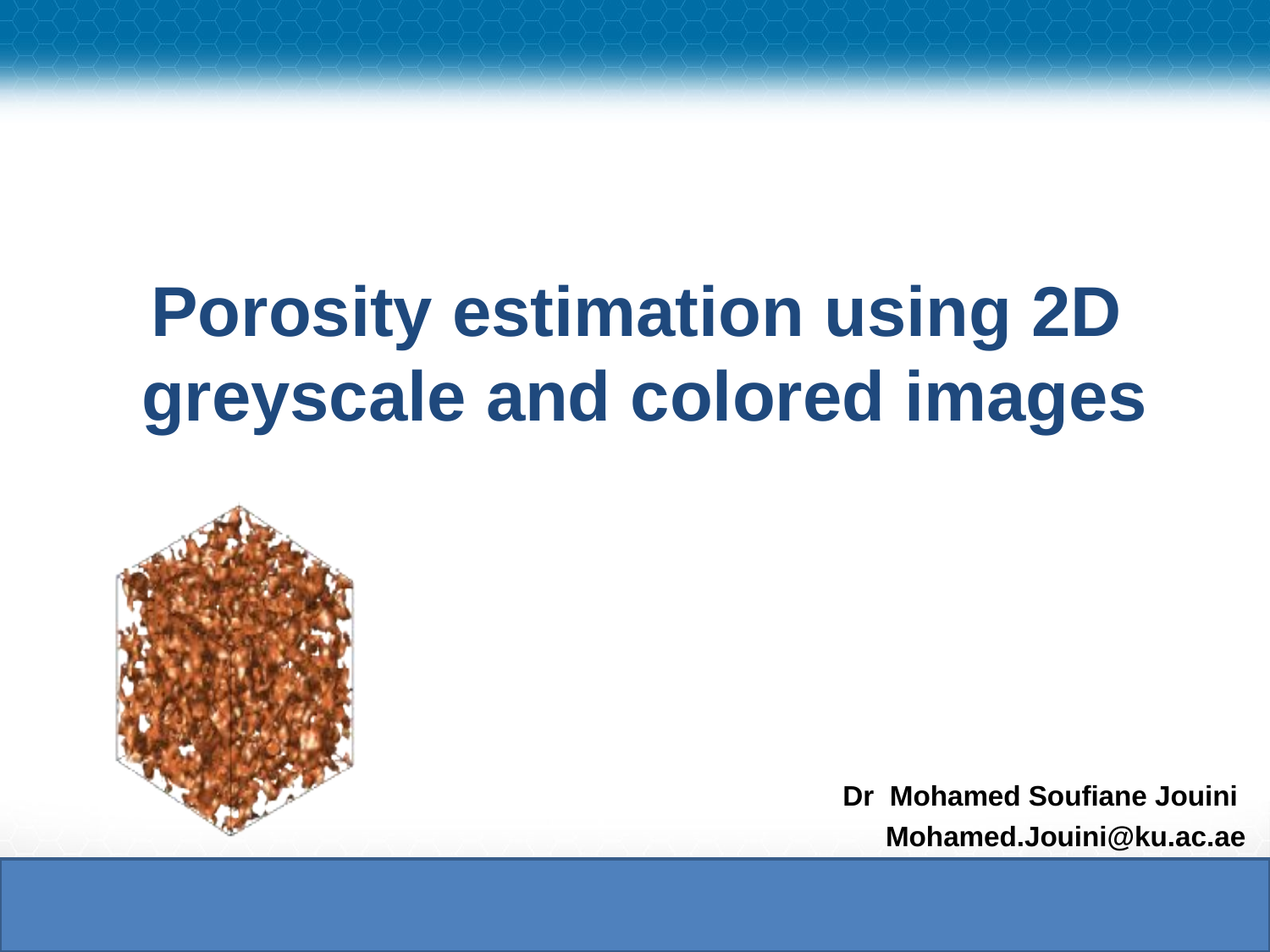

Porosity estimation using 2D greyscale and colored images
				Dr Mohamed Soufiane Jouini
Mohamed.Jouini@ku.ac.ae

## Slide 2
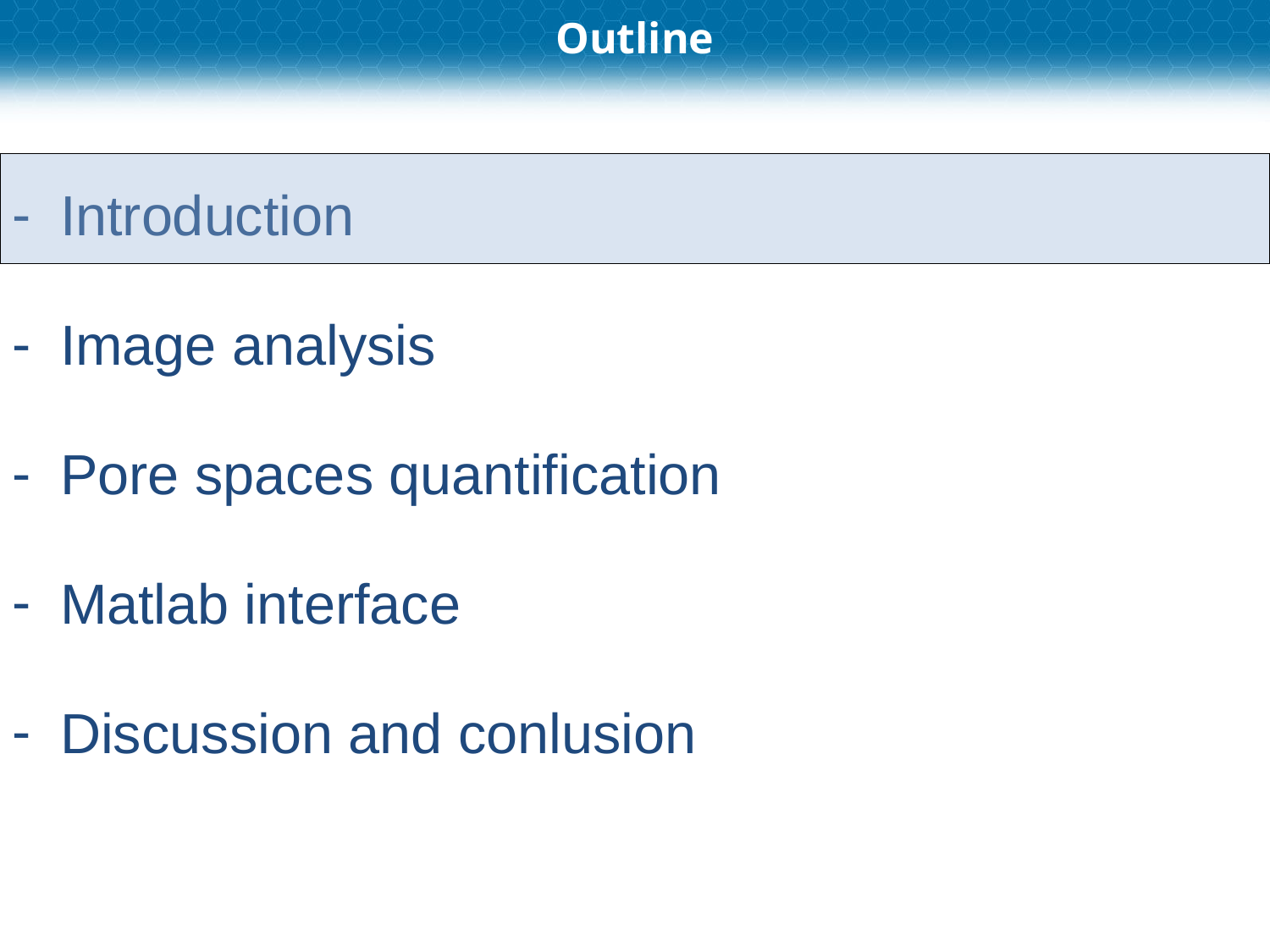

Outline
# Introduction
Image analysis
Pore spaces quantification
Matlab interface
Discussion and conlusion
<number>

## Slide 3
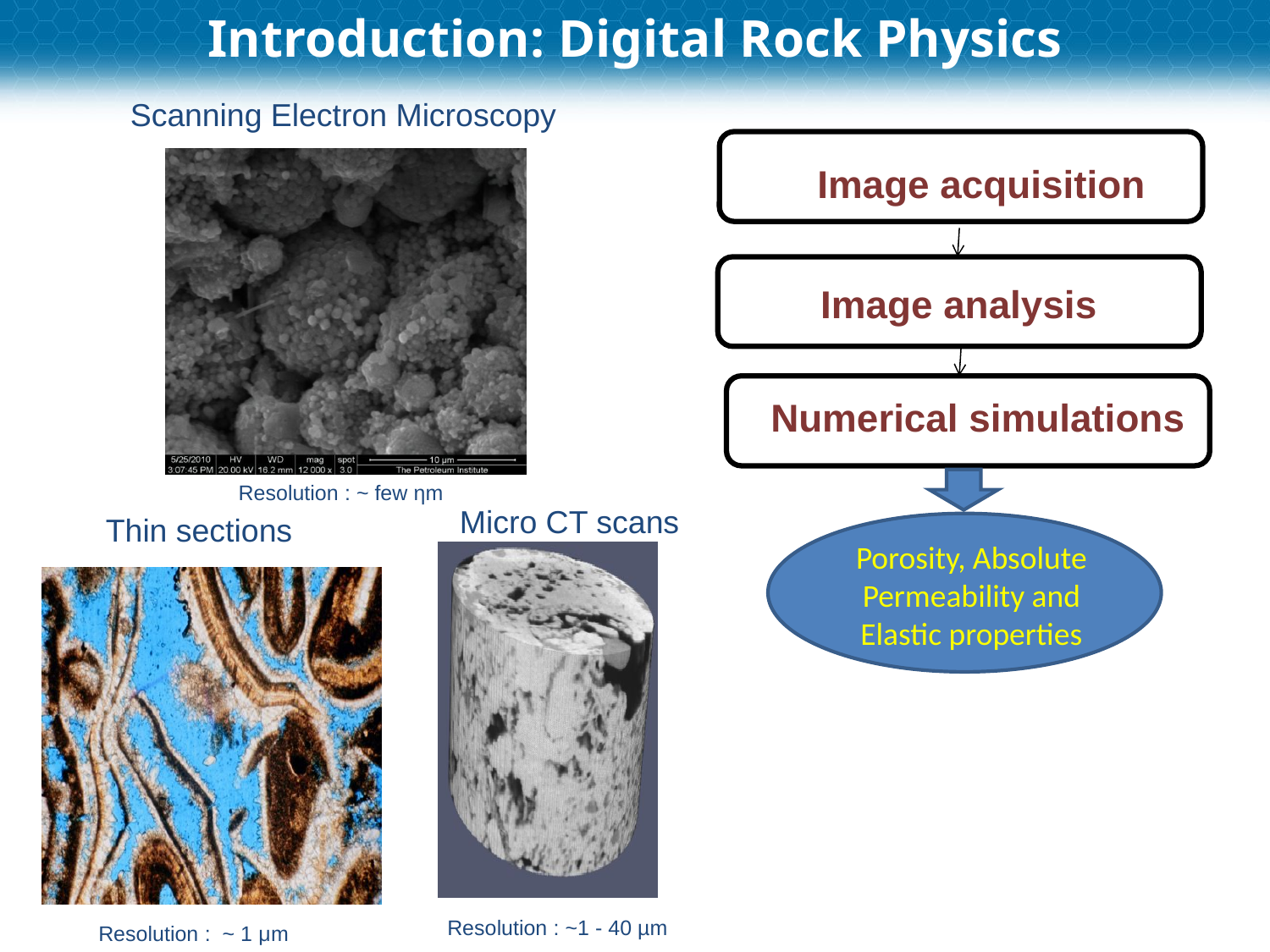

Introduction: Digital Rock Physics
Scanning Electron Microscopy
 Resolution : ~ few ηm
Image acquisition
Image analysis
Numerical simulations
Micro CT scans
Resolution : ~1 - 40 µm
Thin sections
Resolution : ~ 1 μm
Porosity, Absolute Permeability and Elastic properties

## Slide 4
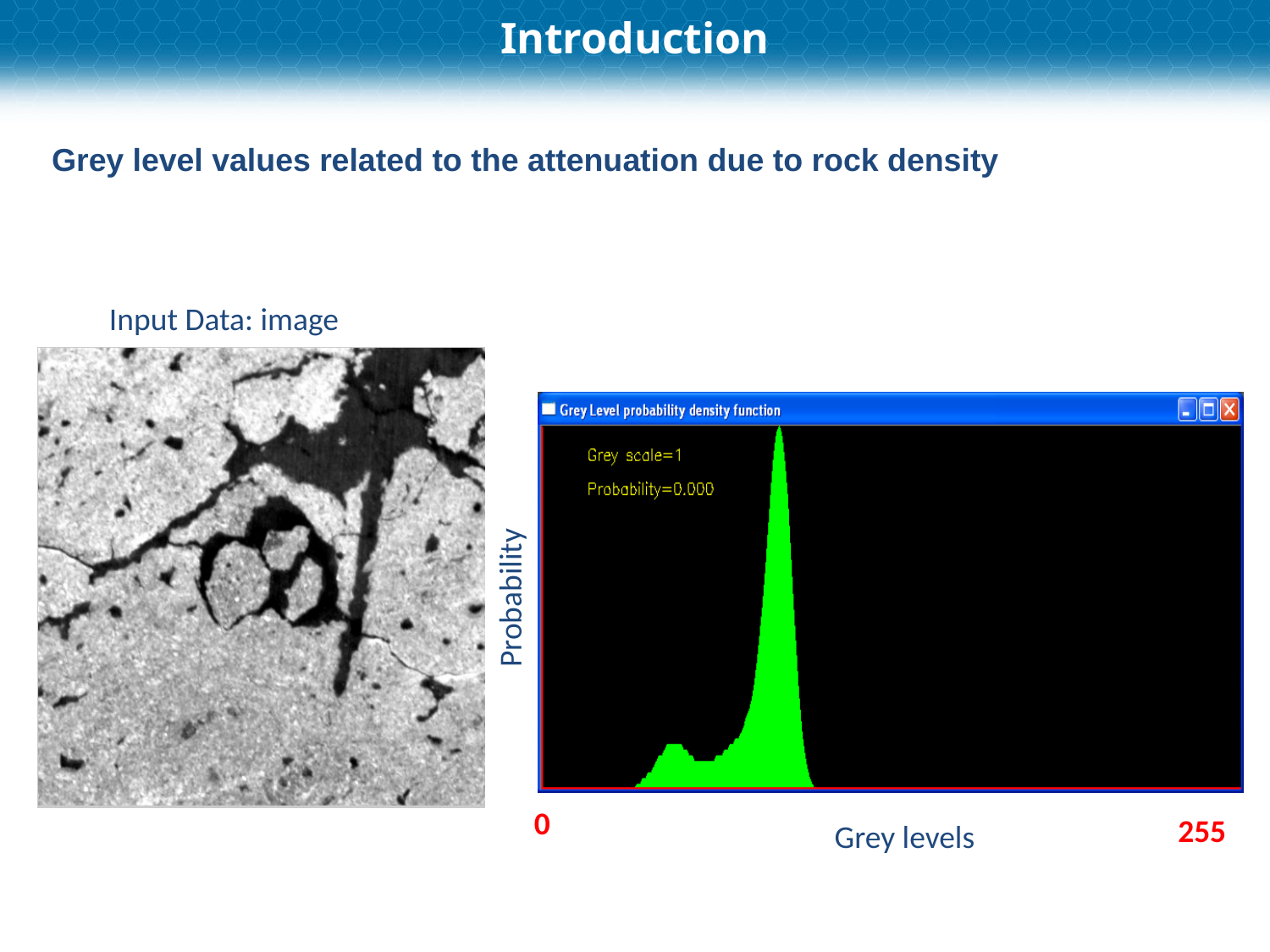

Introduction
Grey level values related to the attenuation due to rock density
Input Data: image
Probability
0
255
Grey levels

## Slide 5
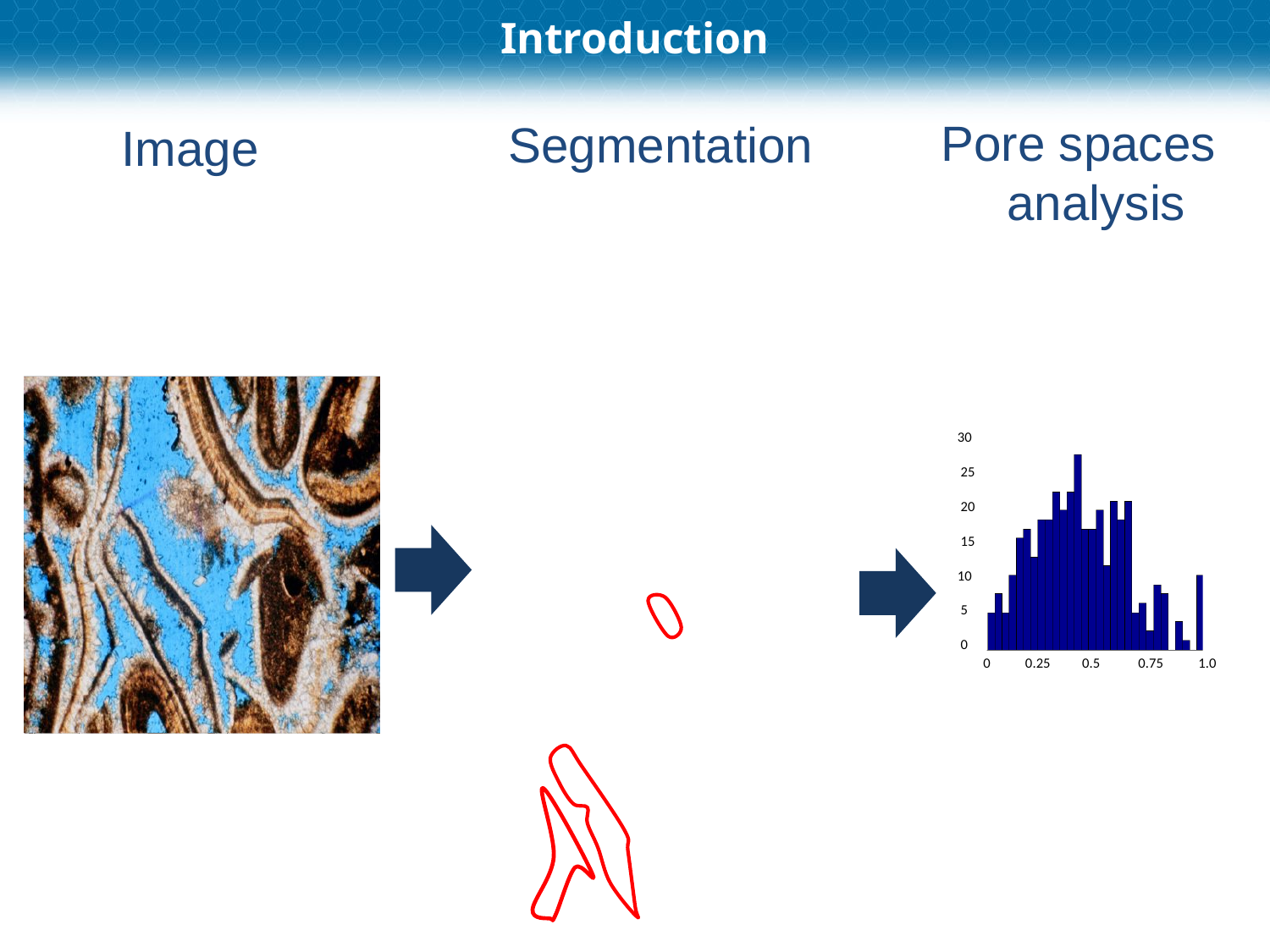

Introduction
Pore spaces analysis
Segmentation
Image
Pores
30
 25
 20
 15
10
 5
 0
Grains
 0 0.25 0.5 0.75 1.0

## Slide 6
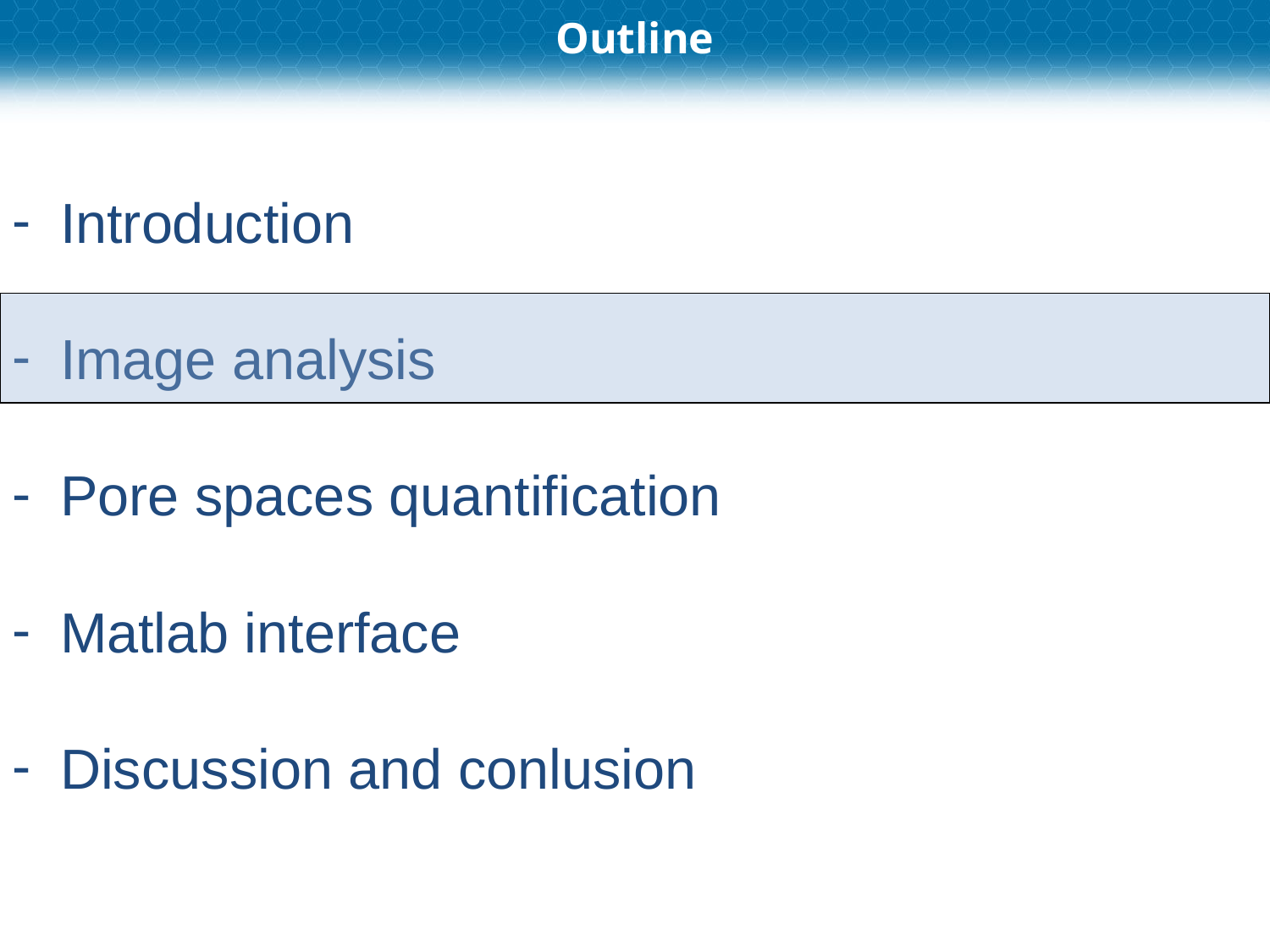

Outline
# Introduction
Image analysis
Pore spaces quantification
Matlab interface
Discussion and conlusion
<number>

## Slide 7
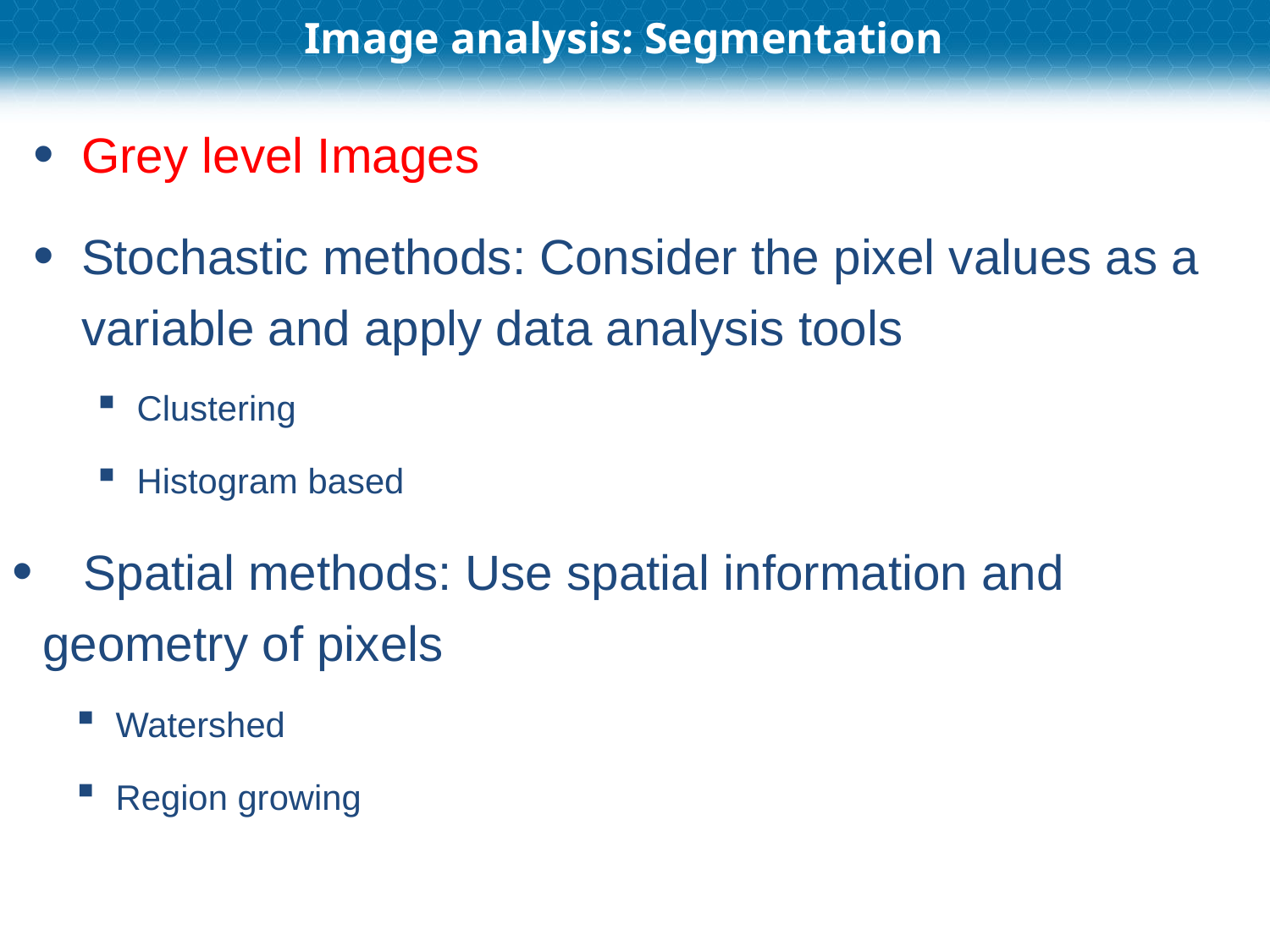

Image analysis: Segmentation
Grey level Images
Stochastic methods: Consider the pixel values as a variable and apply data analysis tools
Clustering
Histogram based
 Spatial methods: Use spatial information and geometry of pixels
Watershed
Region growing

## Slide 8
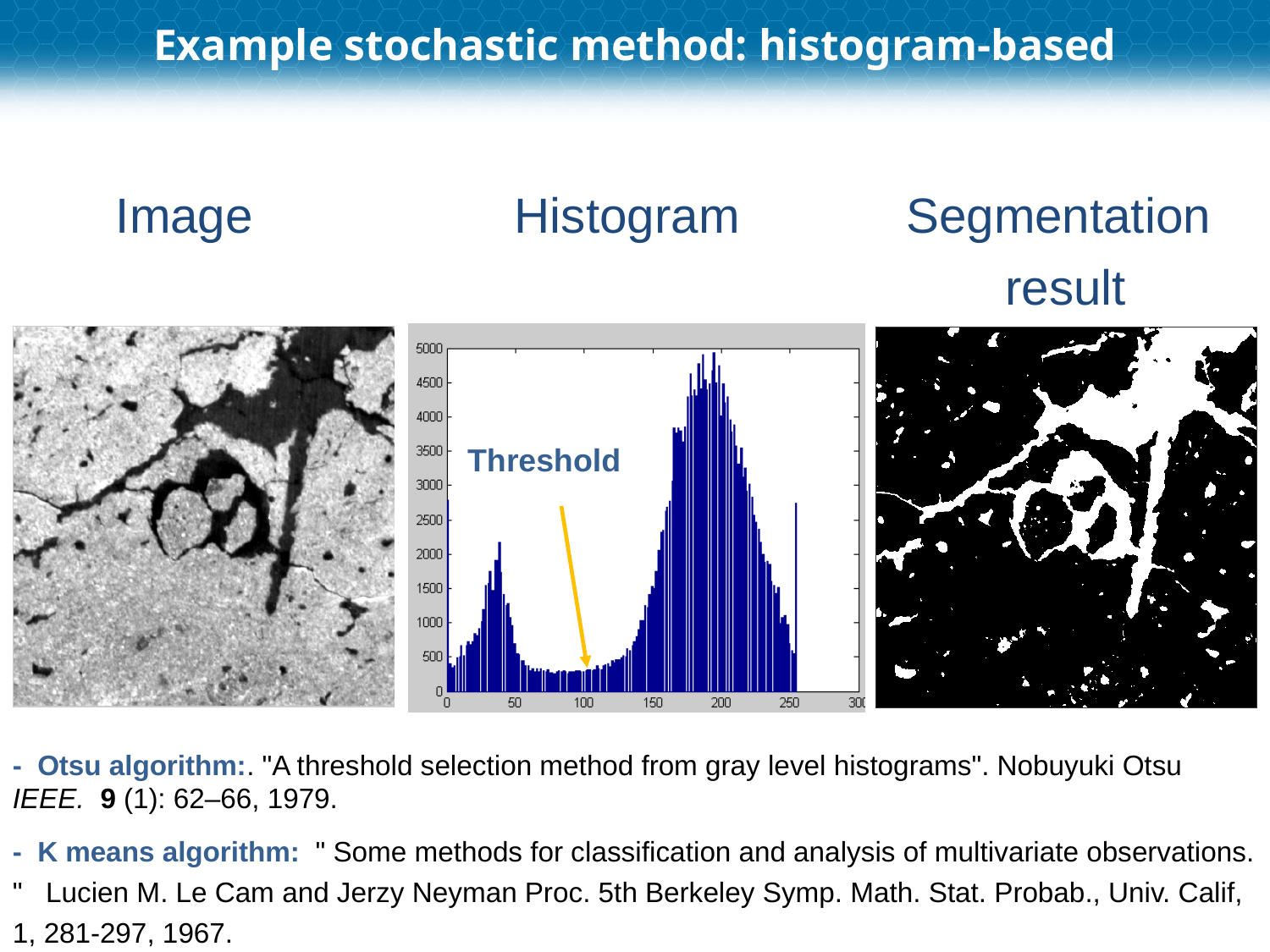

Example stochastic method: histogram-based
Image
Histogram
Segmentation
 result
Threshold
- Otsu algorithm:. "A threshold selection method from gray level histograms". Nobuyuki Otsu IEEE.  9 (1): 62–66, 1979.
- K means algorithm: " Some methods for classification and analysis of multivariate observations. "   Lucien M. Le Cam and Jerzy Neyman Proc. 5th Berkeley Symp. Math. Stat. Probab., Univ. Calif, 1, 281-297, 1967.

## Slide 9
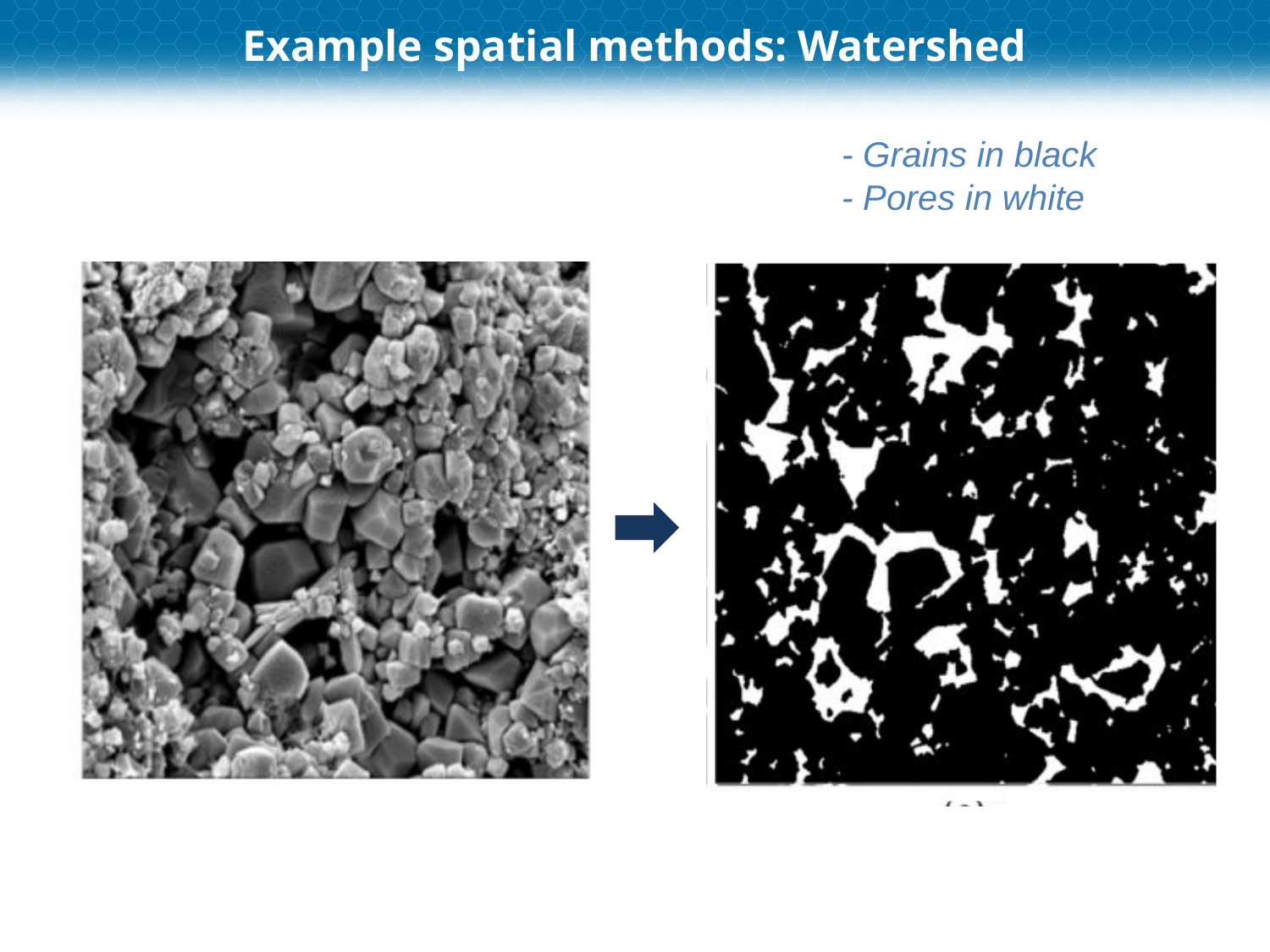

Example spatial methods: Watershed
- Grains in black
- Pores in white

## Slide 10
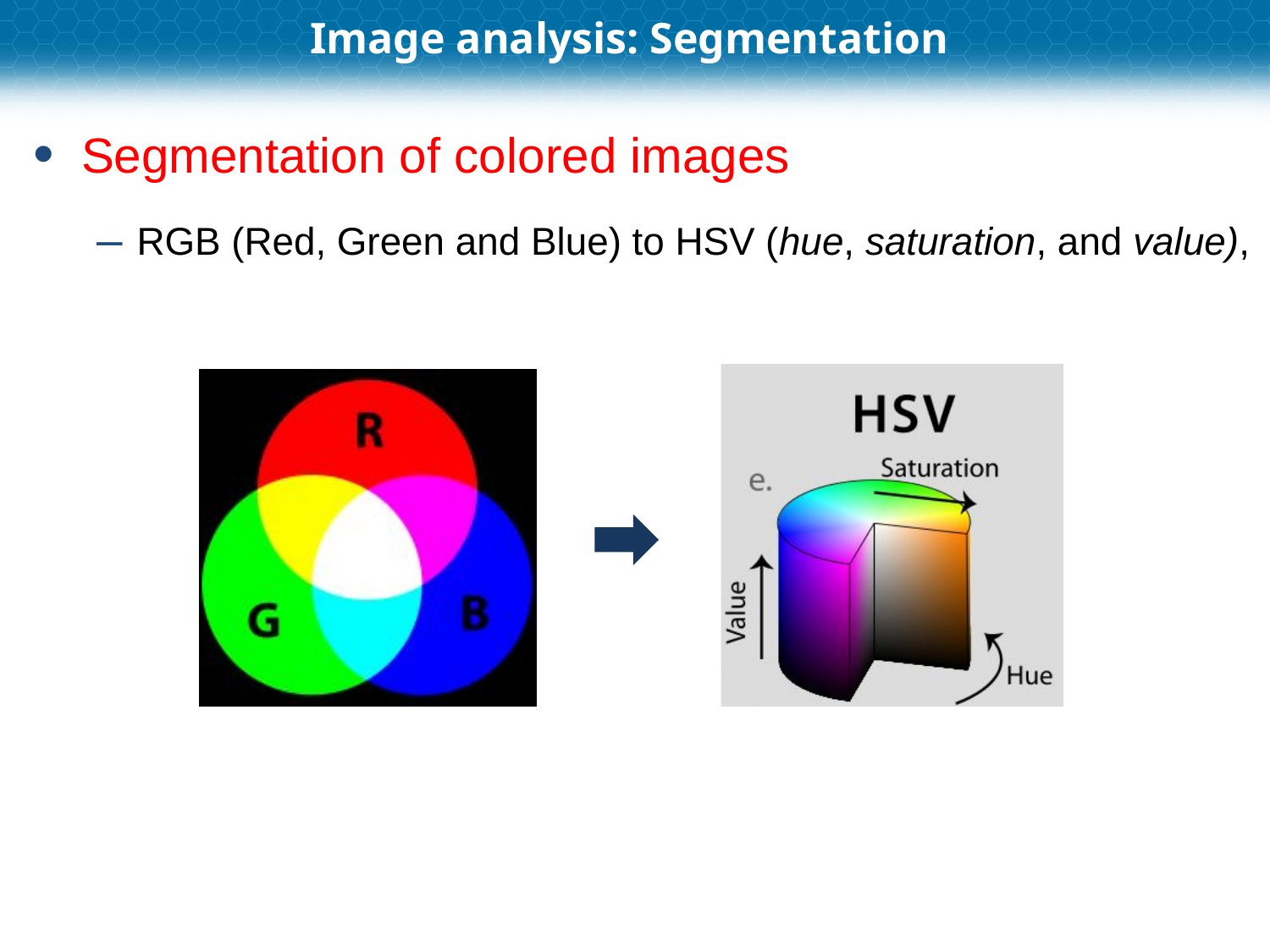

Image analysis: Segmentation
Segmentation of colored images
RGB (Red, Green and Blue) to HSV (hue, saturation, and value),

## Slide 11
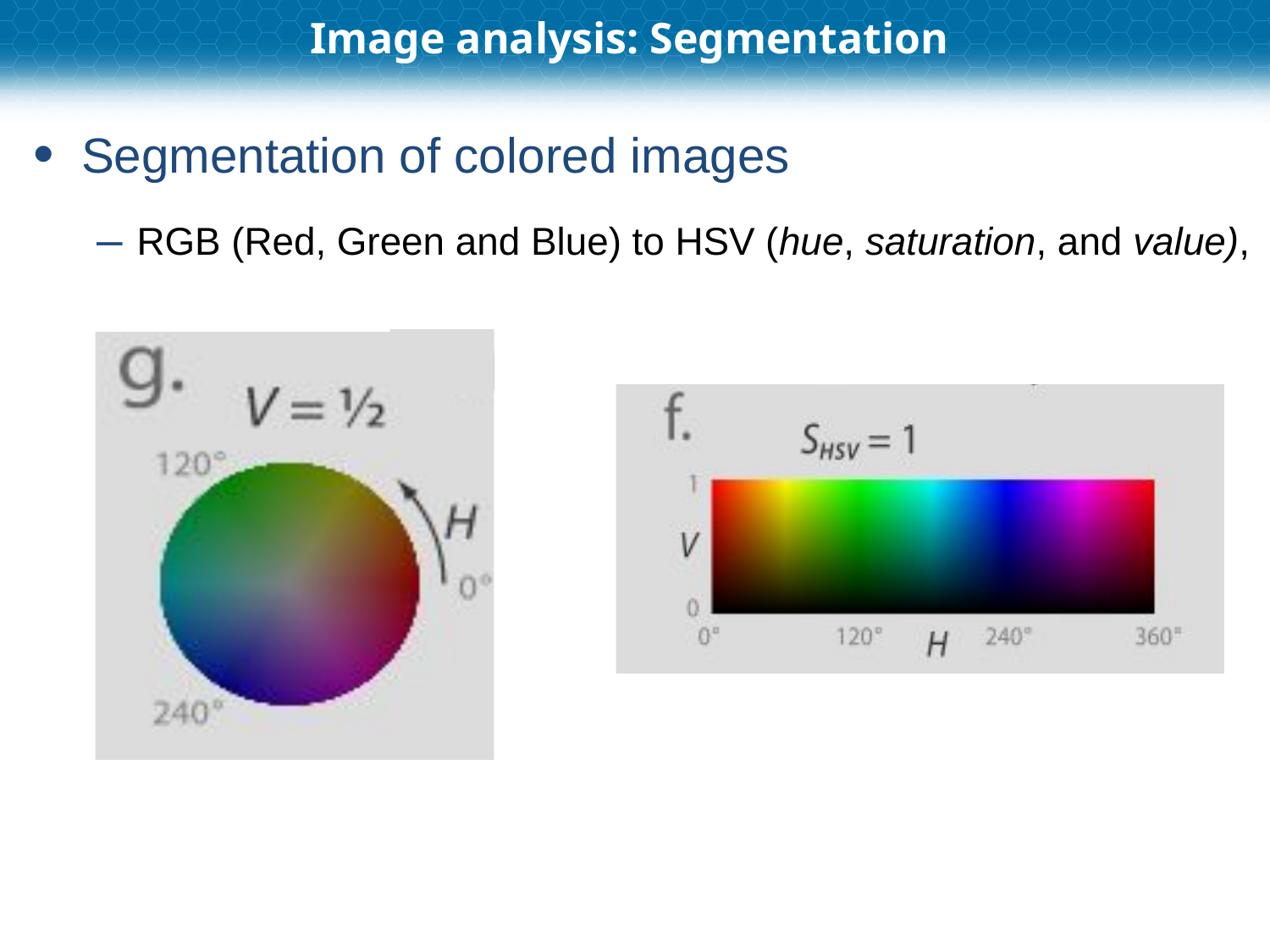

Image analysis: Segmentation
Segmentation of colored images
RGB (Red, Green and Blue) to HSV (hue, saturation, and value),

## Slide 12
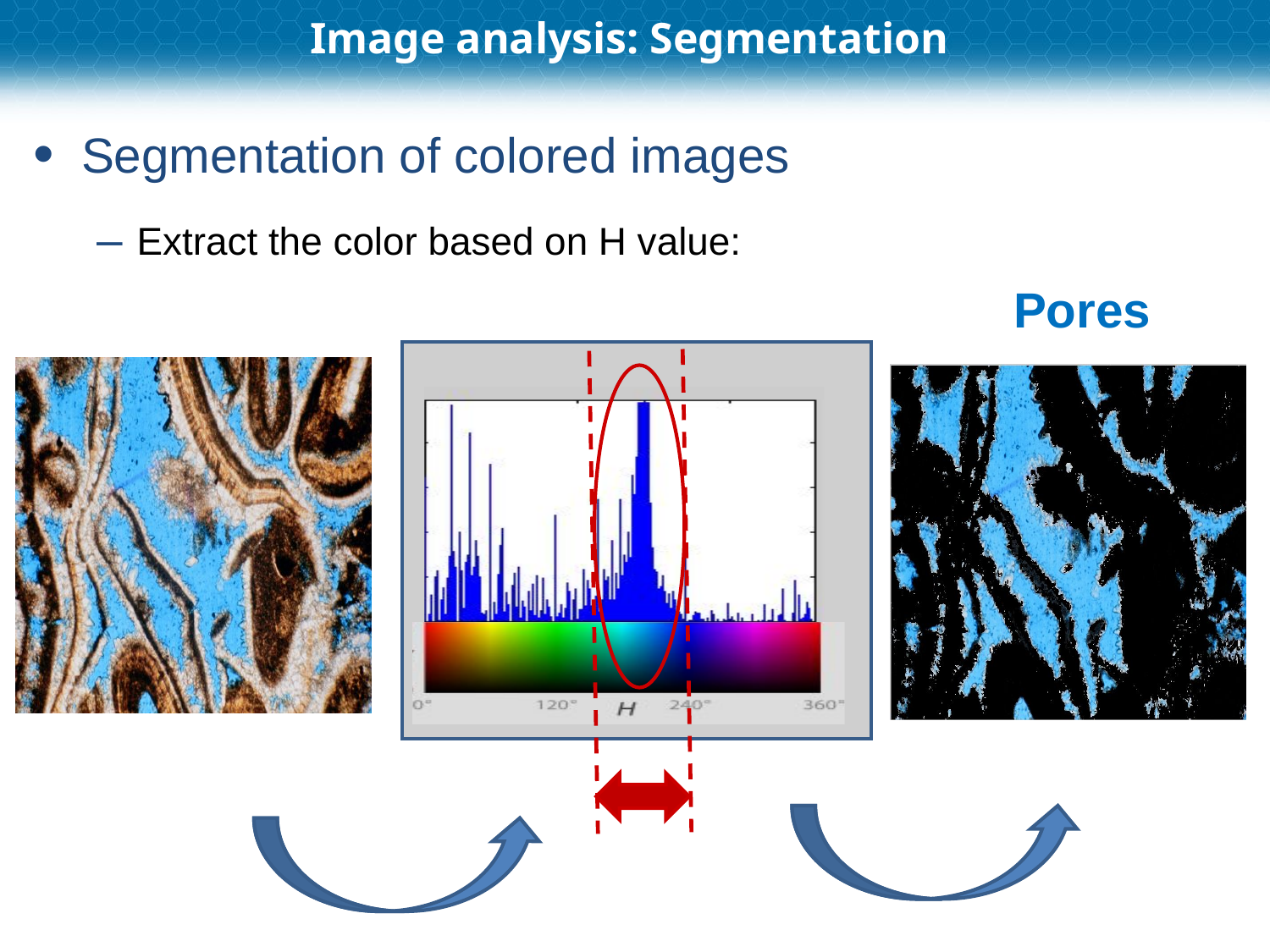

Image analysis: Segmentation
Segmentation of colored images
Extract the color based on H value:
Pores

## Slide 13
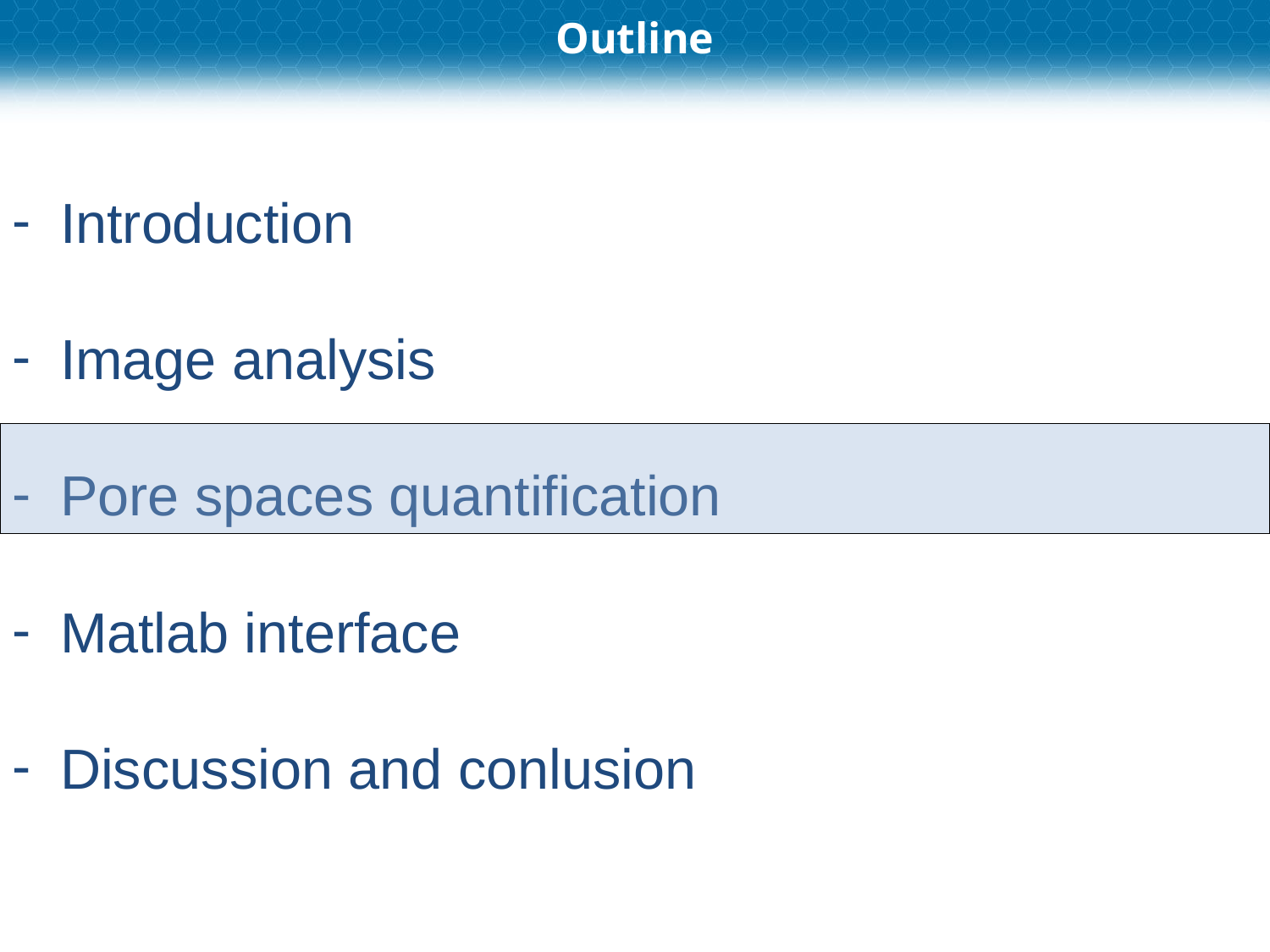

Outline
# Introduction
Image analysis
Pore spaces quantification
Matlab interface
Discussion and conlusion
<number>

## Slide 14
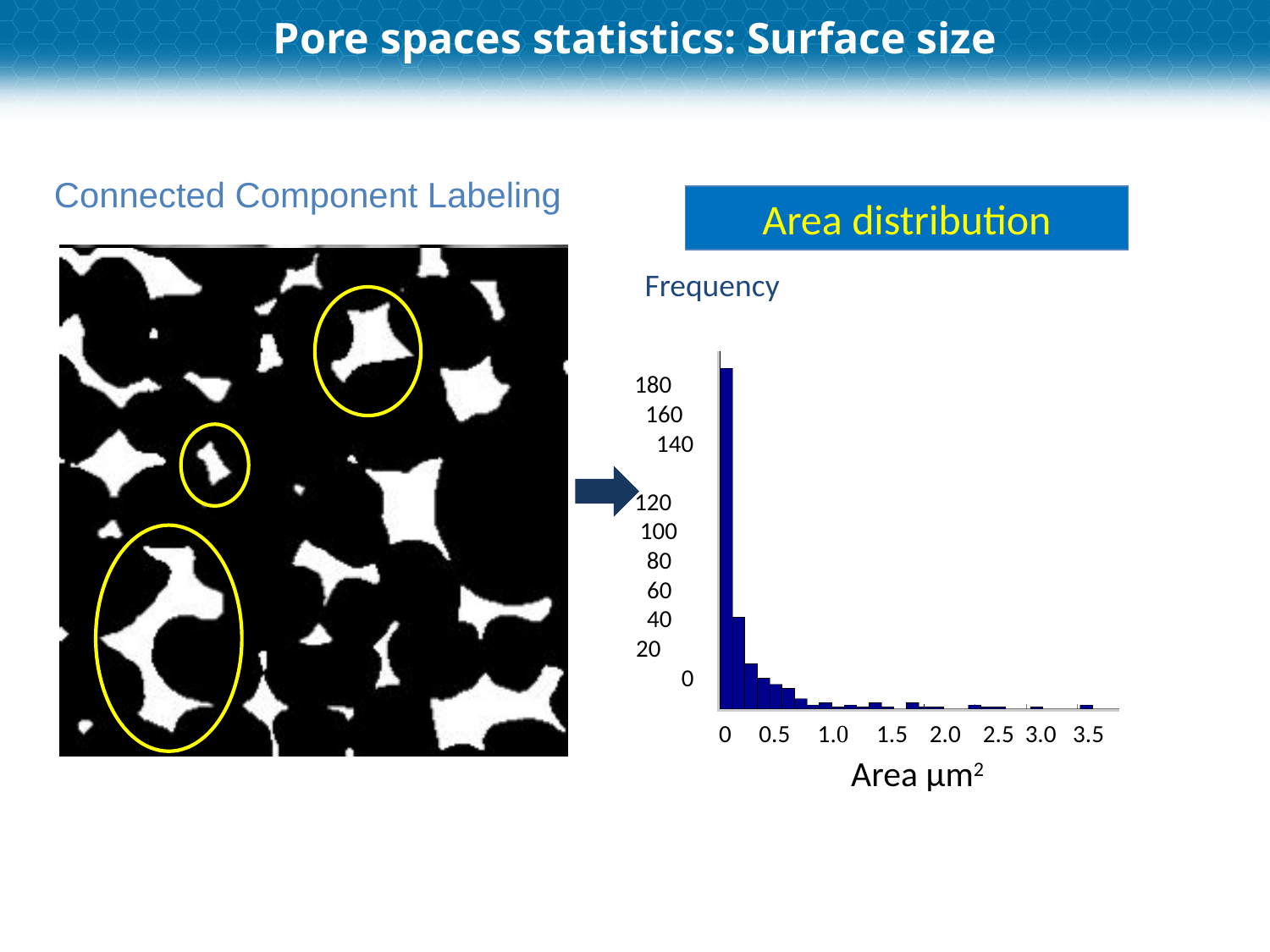

Pore spaces statistics: Surface size
Connected Component Labeling
Area distribution
Frequency
180 160 140 120 100 80 60 40 20 0
0 0.5 1.0 1.5 2.0 2.5 3.0 3.5
Area µm2

## Slide 15
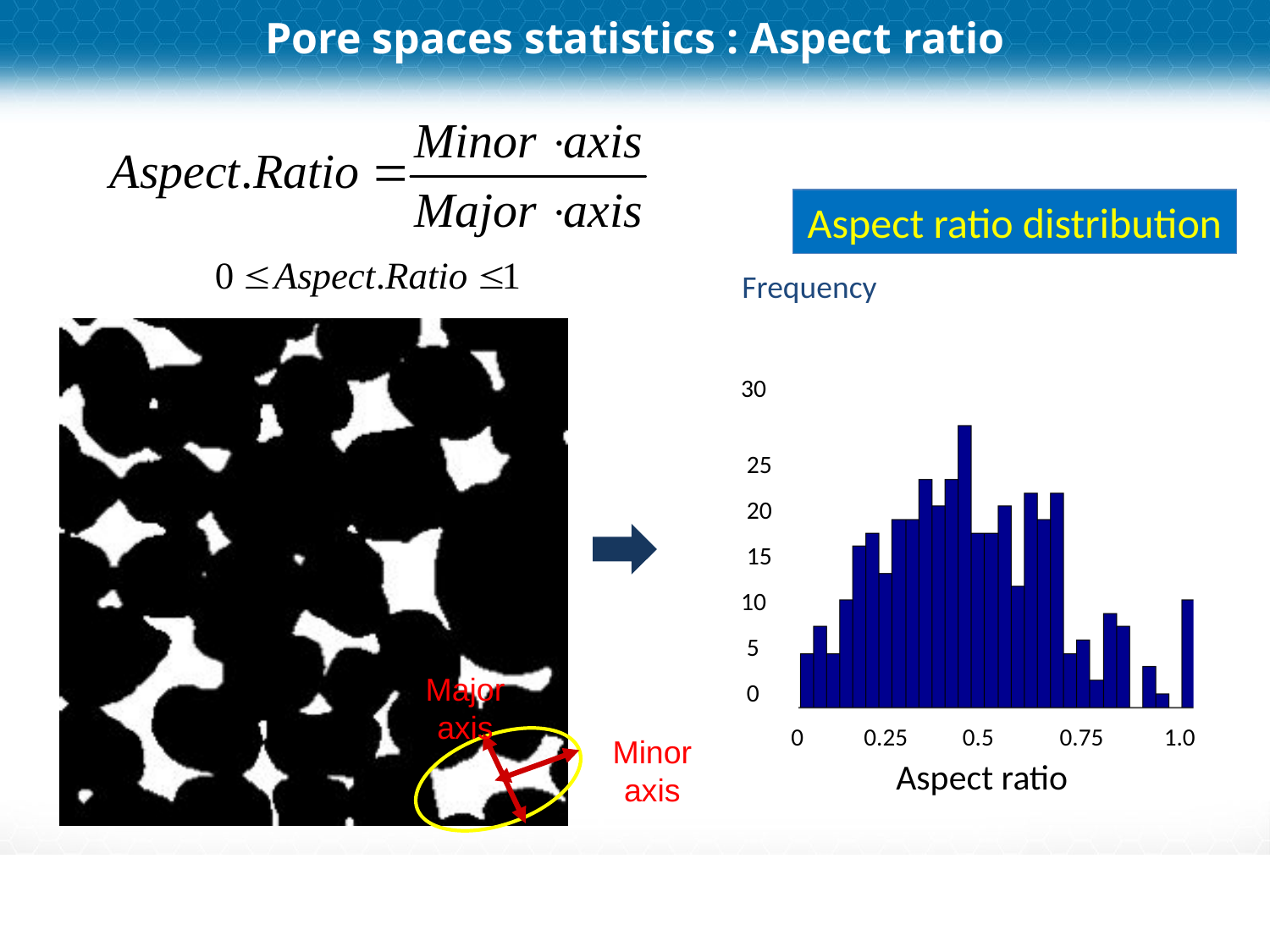

Pore spaces statistics : Aspect ratio
Aspect ratio distribution
Frequency
30
 25
 20
 15
10
 5
 0
Major axis
 0 0.25 0.5 0.75 1.0
Minor axis
Aspect ratio

## Slide 16
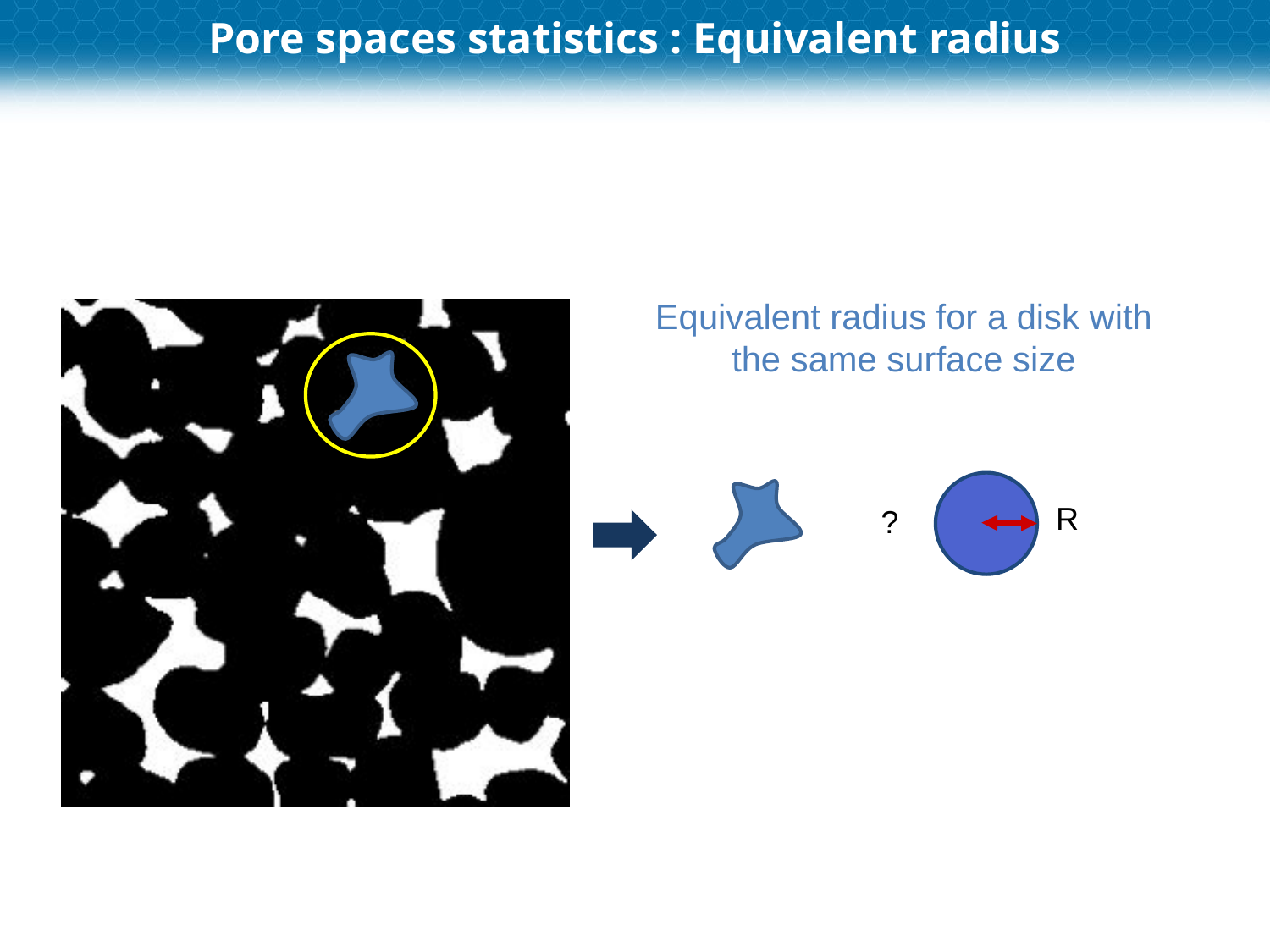

Pore spaces statistics : Equivalent radius
Equivalent radius for a disk with the same surface size
R
?

## Slide 17
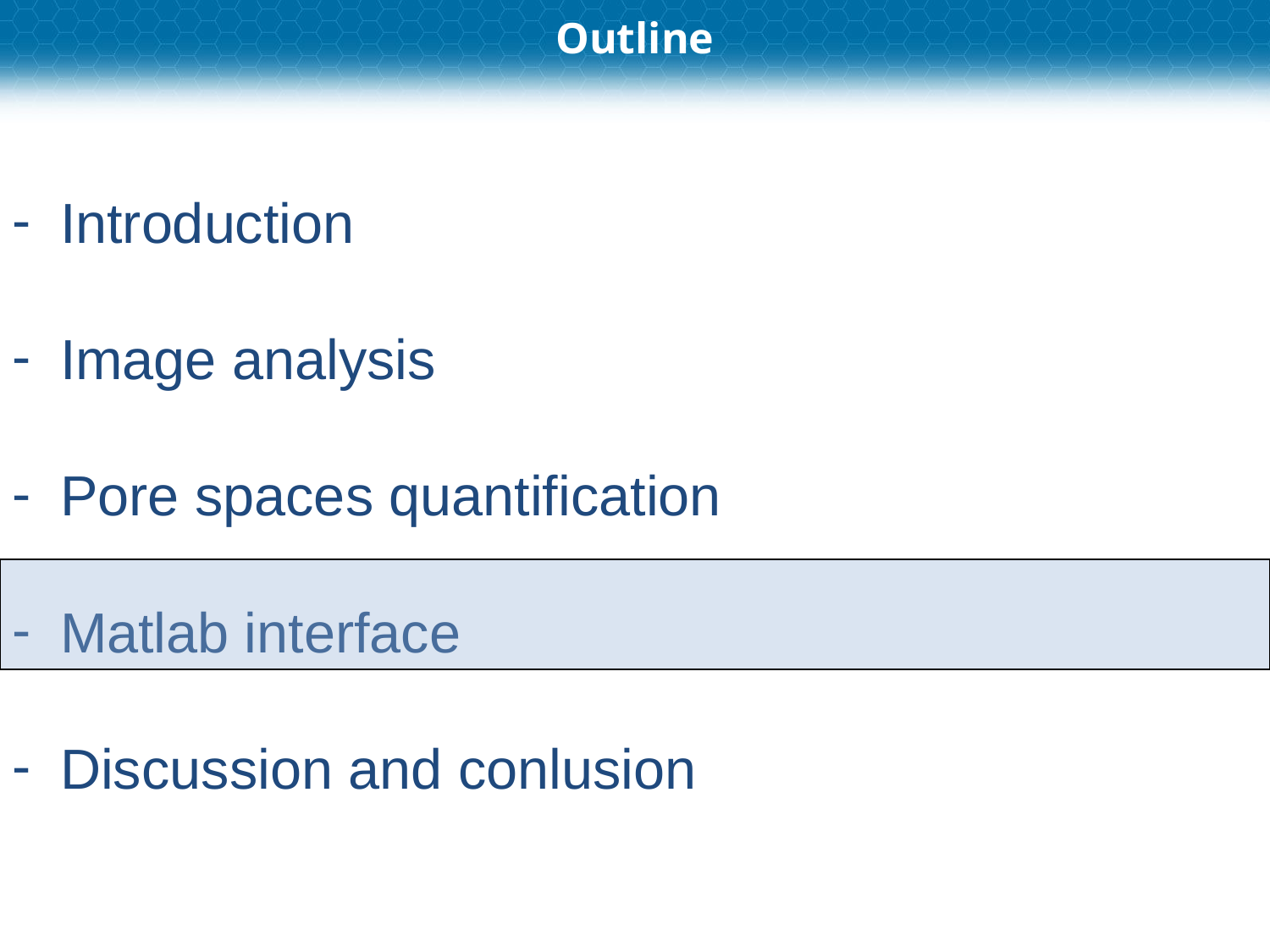

Outline
# Introduction
Image analysis
Pore spaces quantification
Matlab interface
Discussion and conlusion
<number>

## Slide 18
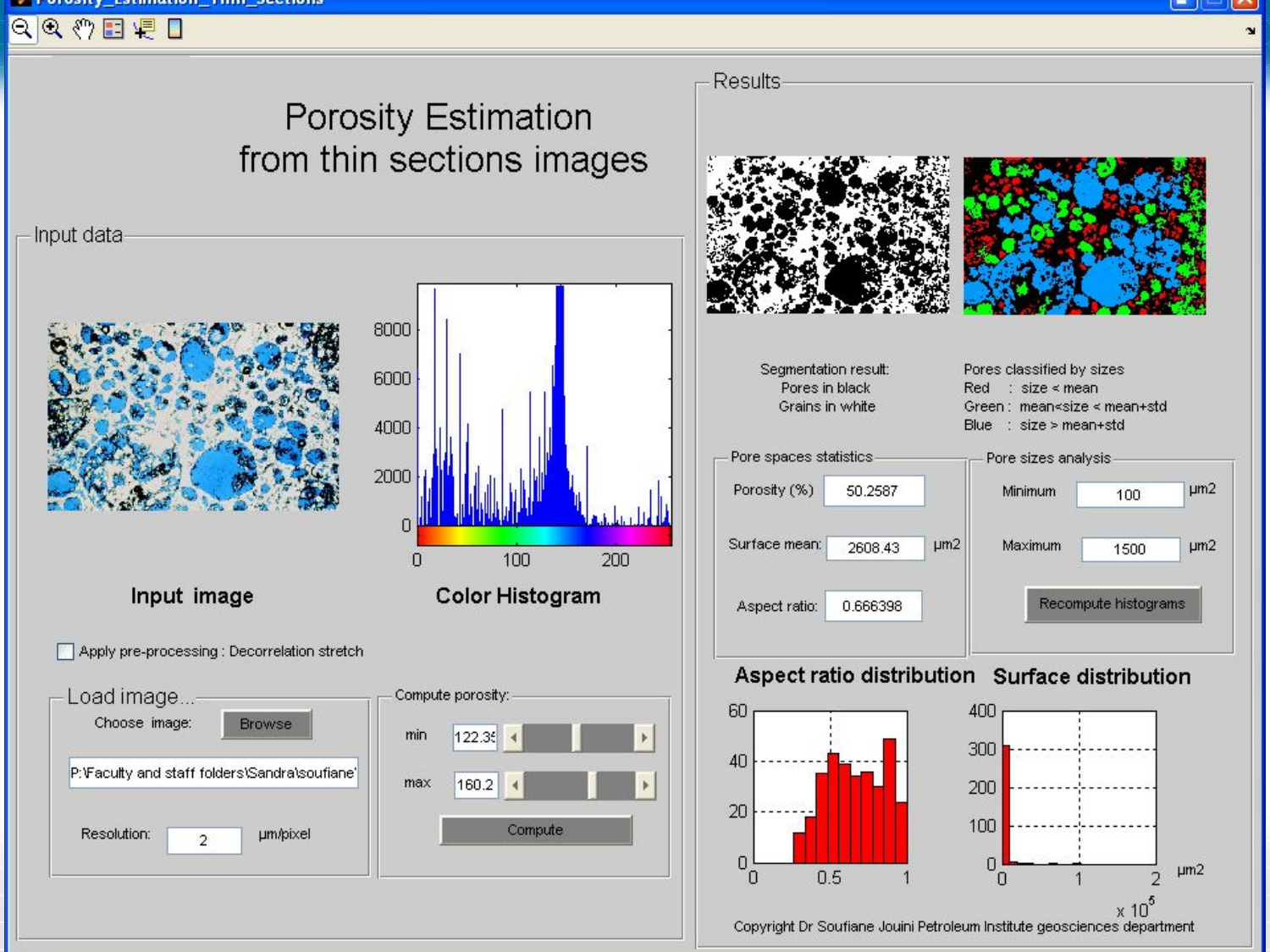

## Slide 19
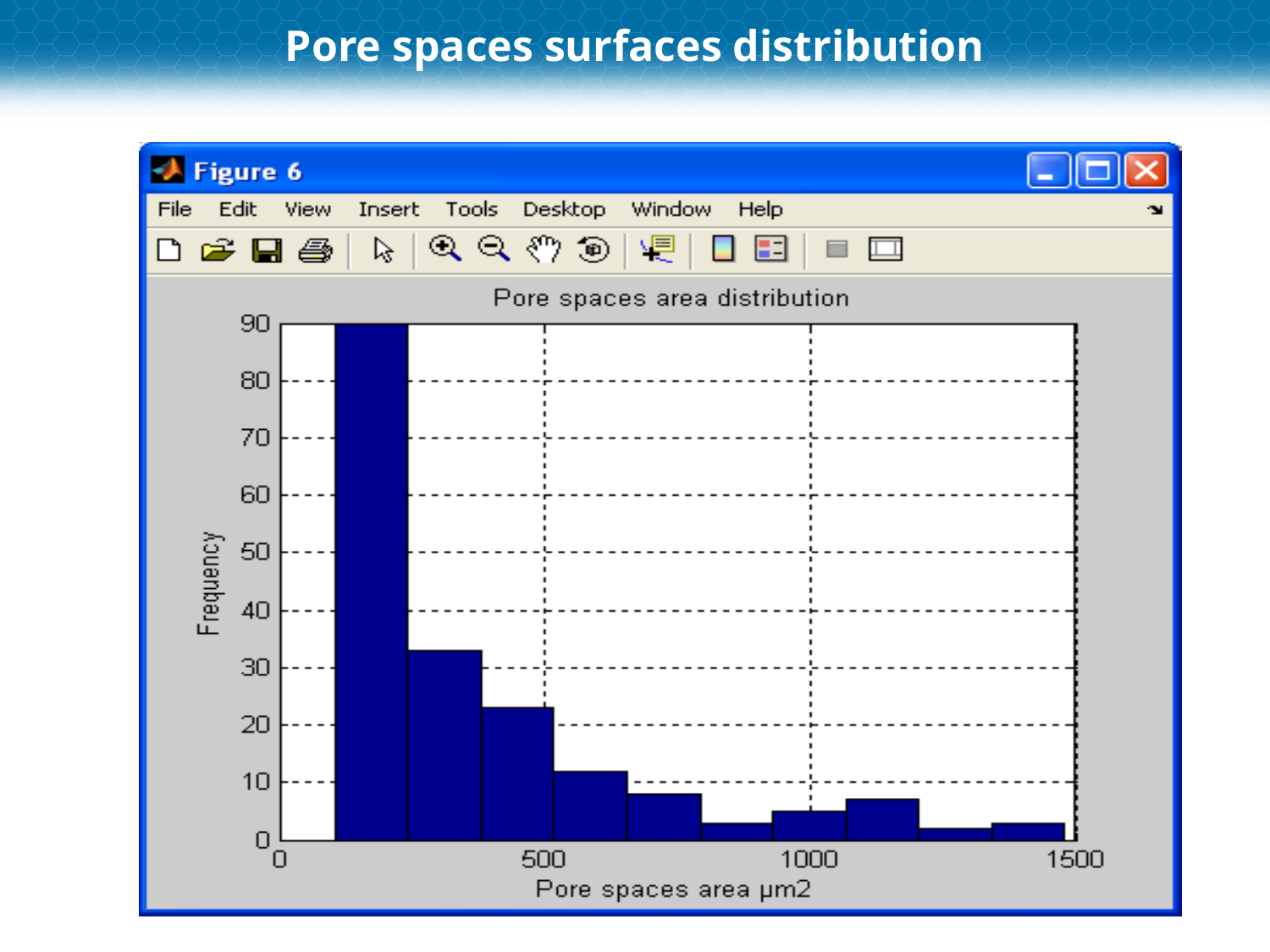

Pore spaces surfaces distribution

## Slide 20
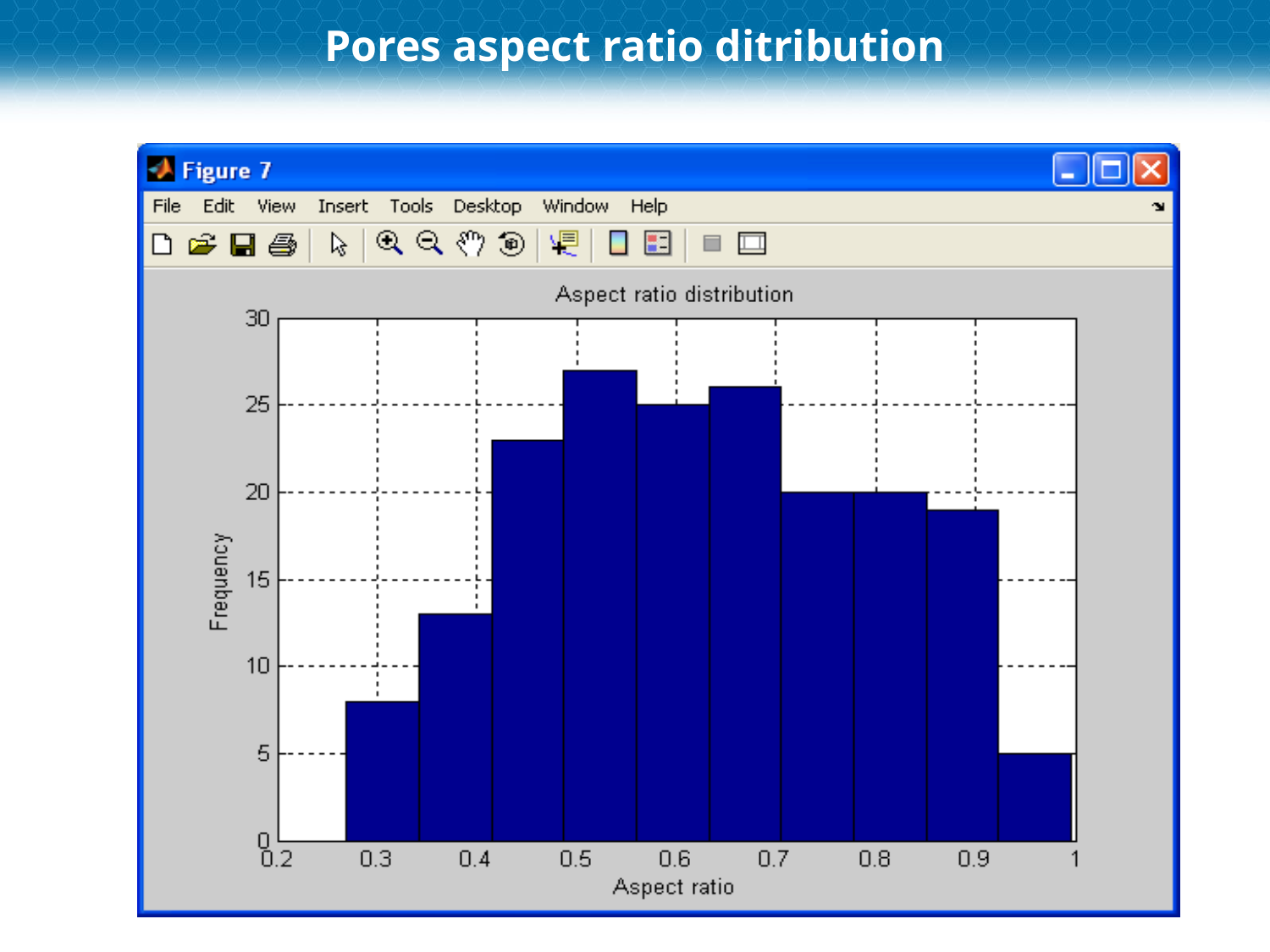

Pores aspect ratio ditribution

## Slide 21
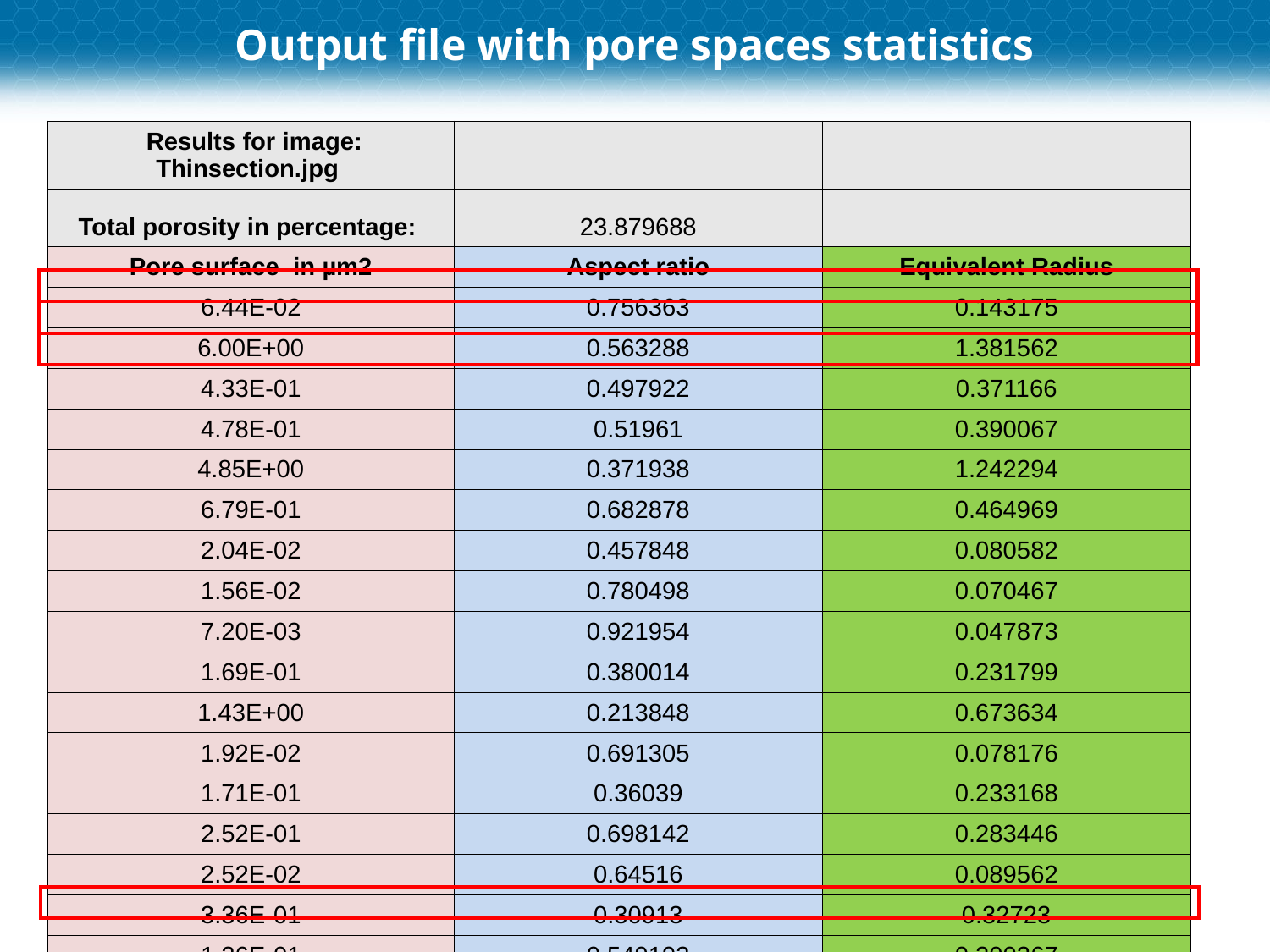

Output file with pore spaces statistics
| Results for image: Thinsection.jpg | | |
| --- | --- | --- |
| Total porosity in percentage: | 23.879688 | |
| Pore surface in µm2 | Aspect ratio | Equivalent Radius |
| 6.44E-02 | 0.756363 | 0.143175 |
| 6.00E+00 | 0.563288 | 1.381562 |
| 4.33E-01 | 0.497922 | 0.371166 |
| 4.78E-01 | 0.51961 | 0.390067 |
| 4.85E+00 | 0.371938 | 1.242294 |
| 6.79E-01 | 0.682878 | 0.464969 |
| 2.04E-02 | 0.457848 | 0.080582 |
| 1.56E-02 | 0.780498 | 0.070467 |
| 7.20E-03 | 0.921954 | 0.047873 |
| 1.69E-01 | 0.380014 | 0.231799 |
| 1.43E+00 | 0.213848 | 0.673634 |
| 1.92E-02 | 0.691305 | 0.078176 |
| 1.71E-01 | 0.36039 | 0.233168 |
| 2.52E-01 | 0.698142 | 0.283446 |
| 2.52E-02 | 0.64516 | 0.089562 |
| 3.36E-01 | 0.30913 | 0.32723 |
| 1.26E-01 | 0.549193 | 0.200267 |
| 9.20E-02 | 0.879374 | 0.171127 |
| 7.20E-03 | 0.921954 | 0.047873 |
| 1.08E-02 | 0.849746 | 0.058632 |
| 3.64E-02 | 0.632142 | 0.107641 |

## Slide 22
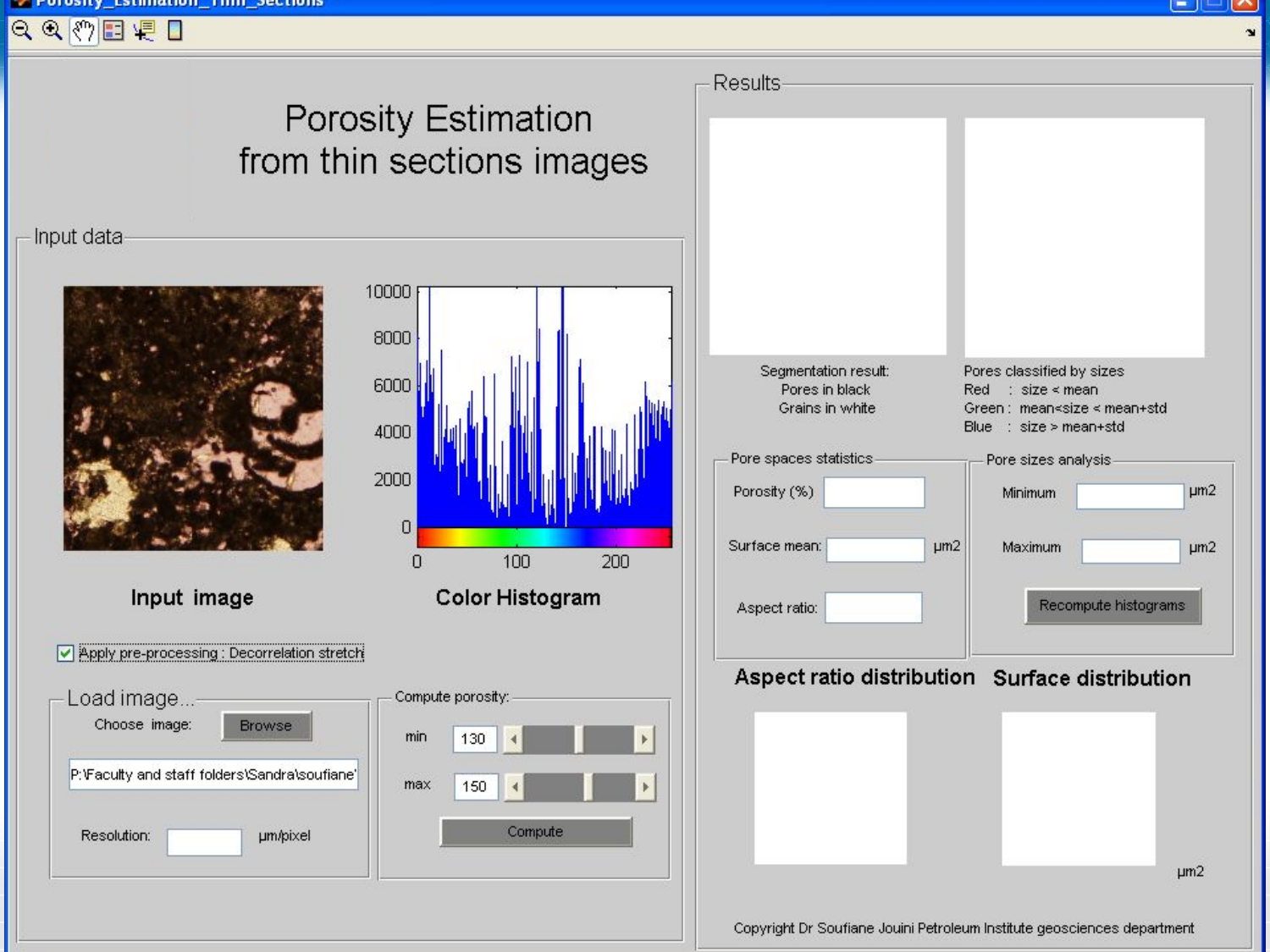

## Slide 23
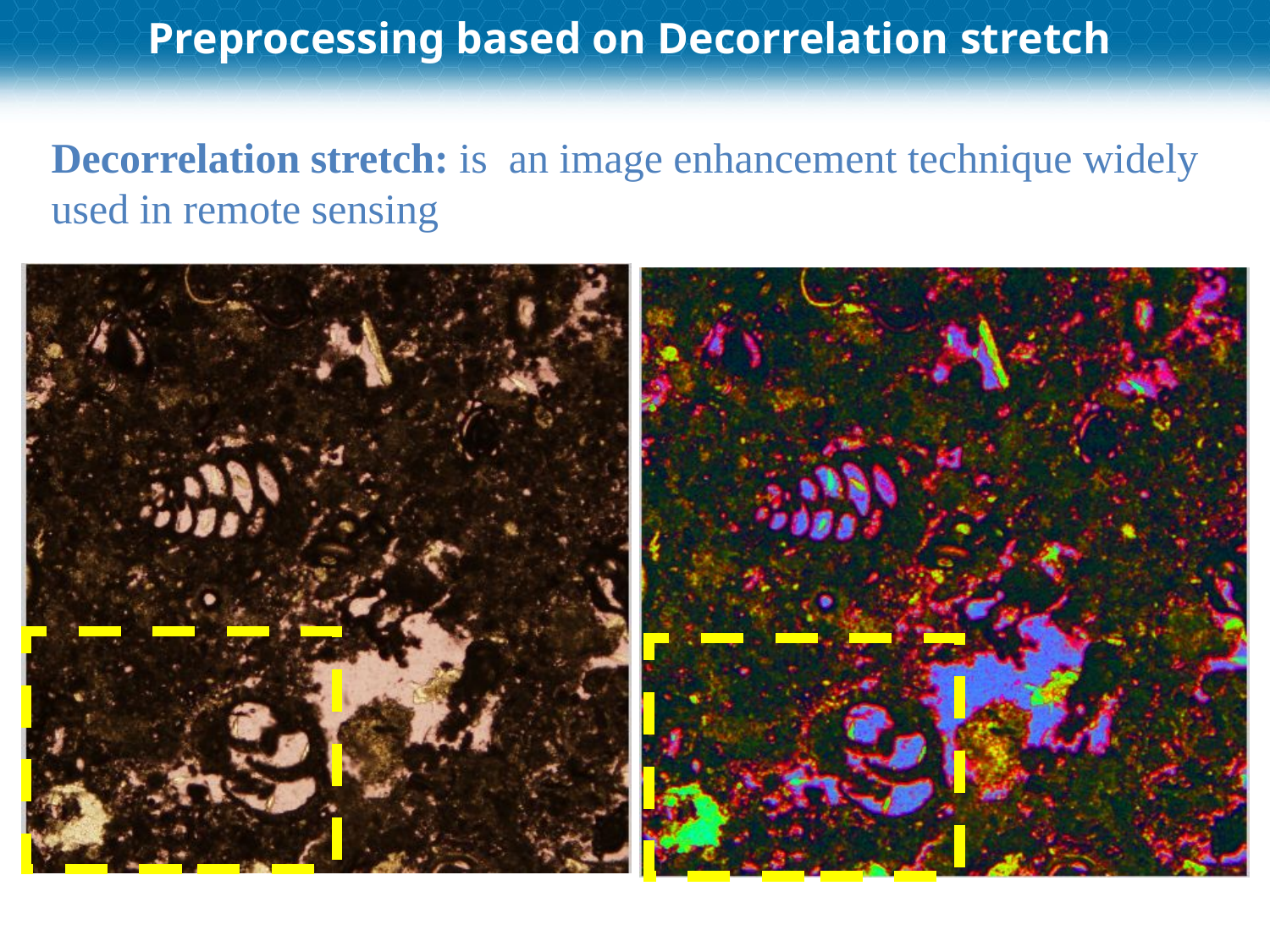

Preprocessing based on Decorrelation stretch
Decorrelation stretch: is an image enhancement technique widely used in remote sensing

## Slide 24
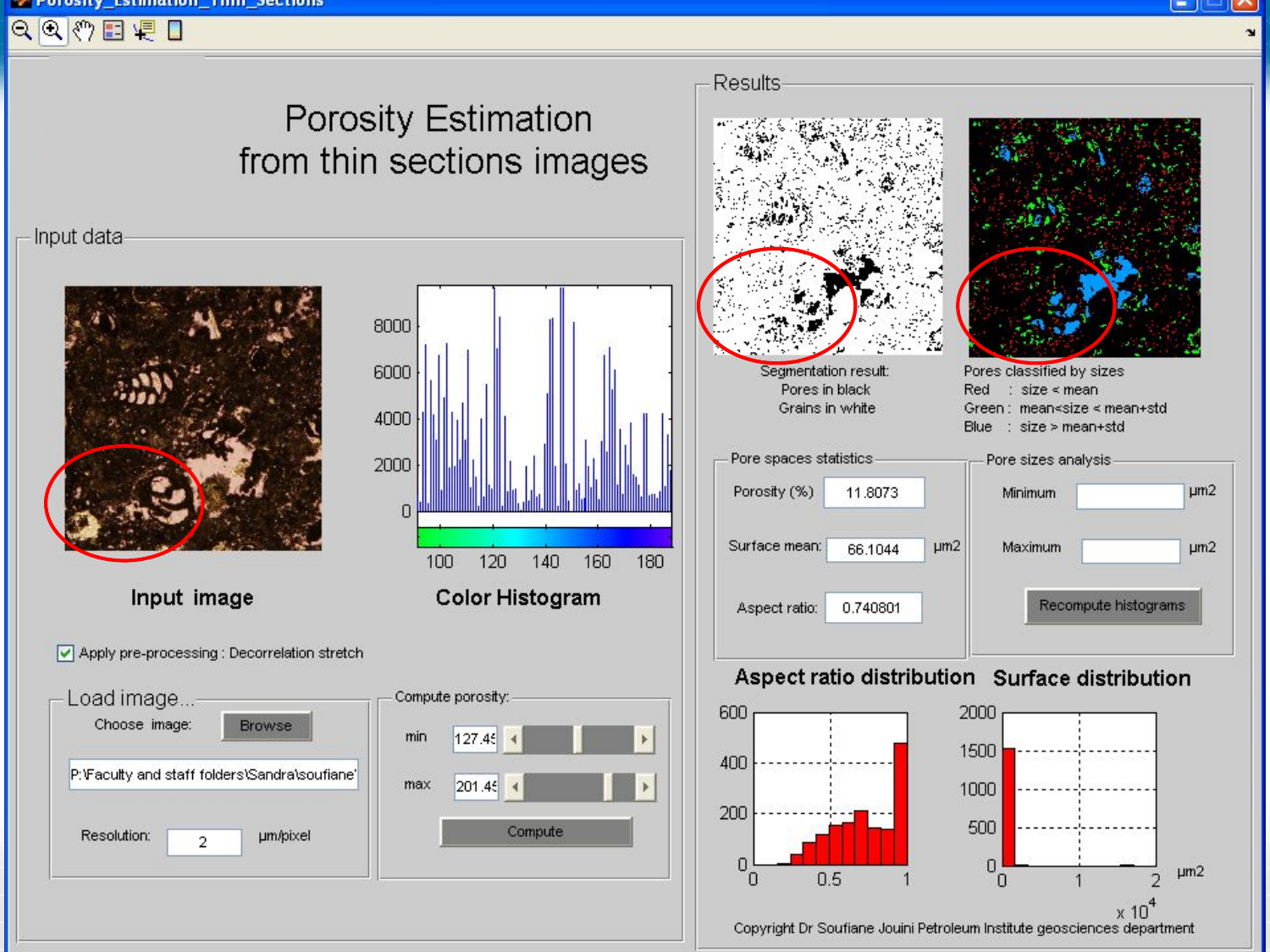

#

## Slide 25
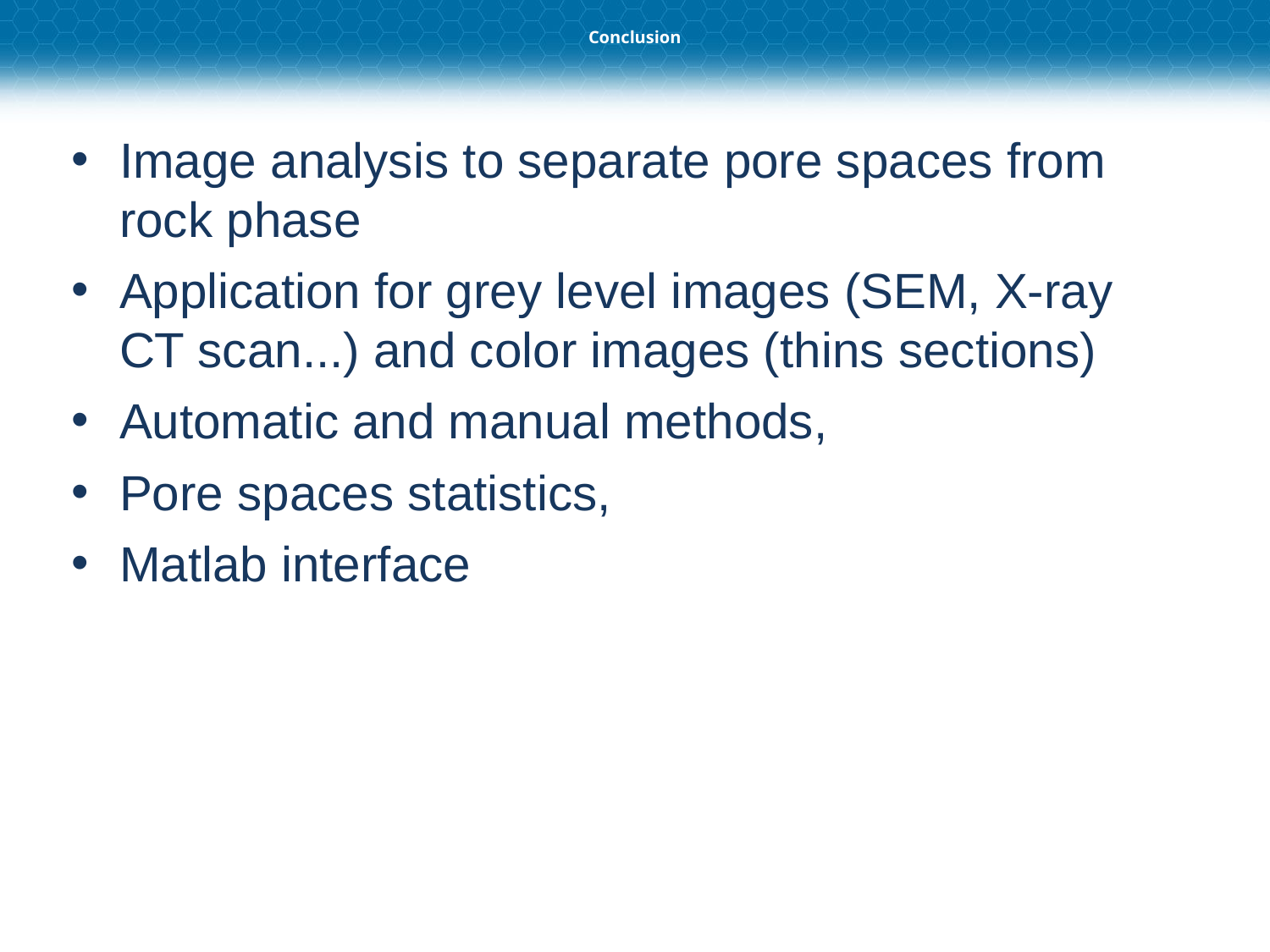

Conclusion
Image analysis to separate pore spaces from rock phase
Application for grey level images (SEM, X-ray CT scan...) and color images (thins sections)
Automatic and manual methods,
Pore spaces statistics,
Matlab interface
